# Mapping microhabitats of lignocellulose decomposition by a microbial consortium

**DOI:** 10.1038/s41589-023-01536-7

**Published:** 2024-02-01

**Authors:** Marija Veličković, Ruonan Wu, Yuqian Gao, Margaret W. Thairu, Dušan Veličković, Nathalie Munoz, Chaevien S. Clendinen, Aivett Bilbao, Rosalie K. Chu, Priscila M. Lalli, Kevin Zemaitis, Carrie D. Nicora, Jennifer E. Kyle, Daniel Orton, Sarai Williams, Ying Zhu, Rui Zhao, Matthew E. Monroe, Ronald J. Moore, Bobbie-Jo M. Webb-Robertson, Lisa M. Bramer, Cameron R. Currie, Paul D. Piehowski, Kristin E. Burnum-Johnson

**Affiliations:** 1 The Environmental Molecular Sciences Laboratory, Pacific Northwest National Laboratory, Richland, WA USA; 2 Biological Sciences Division, Pacific Northwest National Laboratory, Richland, WA USA; 3 Wisconsin Institute for Discovery and Department of Plant Pathology, University of Wisconsin-Madison, Madison, WI USA; 4 Department of Microchemistry, Proteomics, Lipidomics, and Next Generation Sequencing, Genentech, San Francisco, CA USA; 5 Department of Bacteriology, University of Wisconsin-Madison, Madison, WI USA; 6 Department of Biochemistry & Biomedical Sciences, McMaster University, Hamilton, Ontario Canada

**Keywords:** Metabolomics, Microbiology, Imaging, Carbohydrates

## Abstract

The leaf-cutter ant fungal garden ecosystem is a naturally evolved model system for efficient plant biomass degradation. Degradation processes mediated by the symbiotic fungus *Leucoagaricus gongylophorus* are difficult to characterize due to dynamic metabolisms and spatial complexity of the system. Herein, we performed microscale imaging across 12-µm-thick adjacent sections of *Atta cephalotes* fungal gardens and applied a metabolome-informed proteome imaging approach to map lignin degradation. This approach combines two spatial multiomics mass spectrometry modalities that enabled us to visualize colocalized metabolites and proteins across and through the fungal garden. Spatially profiled metabolites revealed an accumulation of lignin-related products, outlining morphologically unique lignin microhabitats. Metaproteomic analyses of these microhabitats revealed carbohydrate-degrading enzymes, indicating a prominent fungal role in lignocellulose decomposition. Integration of metabolome-informed proteome imaging data provides a comprehensive view of underlying biological pathways to inform our understanding of metabolic fungal pathways in plant matter degradation within the micrometer-scale environment.

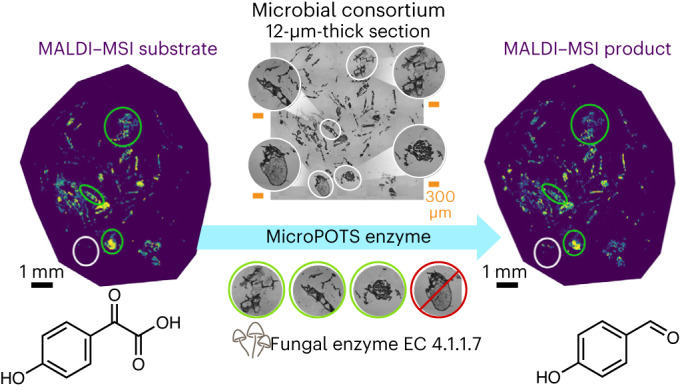

## Abstract (web summary)

Metabolome-informed proteome imaging provides a comprehensive view of underlying biological pathways within micrometer-scale microhabitats of the fungal garden, informing the understanding of metabolic fungal pathways in plant matter degradation.

## Main

Leaf-cutter ants in the genus *Atta* are exemplary herbivores that can consume as much as 17% of the leaf biomass produced in Neotropical forest ecosystems^1^. Although worker ants can use some nutrients from plant sap as they forage for fresh plant material^2–4^, fungus-growing attine ants gain access to nutrients primarily by cultivating a symbiotic fungus, *Leucoagaricus gongylophorus*, on fresh leaves in specialized underground structures called fungal gardens. Fungal gardens host other microbial symbionts, including nitrogen-fixing bacteria that provide both ants and fungi with nitrogen^5^. Other resident bacterial community members help the fungus degrade plant biomass by producing amino acids and vitamins, enabling the fungus to thrive^6^. Within the fungal garden, there is a vertical gradient of plant matter degradation, and differences in the abundance of *L. gongylophorus* key structures can also be observed. These differences are characterized by a distinct visual appearance at each stratum and their unique molecular properties^7–9^. The middle stratum of the fungal garden is characterized by increased fungal hyphal growth and gongylidia (specialized hyphal swellings), which the ants consume^4,10^. To investigate the spatial organization and lignocellulose degradation activities of *L. gongylophorus* in this ecosystem, our study targeted the middle section of the fungal garden.

Mass spectrometry (MS) studies on the fungal gardens of leaf-cutter ants have provided a glimpse into the molecular roles of microbial members within these ecosystems^6,8,11–14^. However, these previous studies profiled molecules from bulk fungal garden samples, thereby averaging the biological processes across the ecosystem and masking their spatial localization, the biological origin of metabolites and their molecular dynamics^6–8,11^. Complex environmental ecosystems, such as these fungal gardens, contain microhabitats with different species, resources and activities. Using bulk proteomic and metabolomic measurements to characterize these gardens averages the molecular signatures from ants, plants and diverse bacterial and fungal species. This averaging creates noise in MS data and dilutes the low-abundance pathways of interest, often making them undetectable. The ability to provide a snapshot of spatially resolved molecules within microhabitats of complex ecosystems and trace a natural compound back to its organism of origin are of enormous value in elucidating biological mechanisms occurring within multiorganismal systems.

MS imaging (MSI) provides untargeted in situ mapping of molecular distributions across biological samples^15–20^. Matrix-assisted laser desorption/ionization–MSI (MALDI–MSI) has been applied to profile the metabolic capacity of microbial communities^21–23^ and detect the degradation of highly complex plant-associated products, such as lignin^24,25^. Previous MALDI–Fourier transform ion cyclotron resonance–MS (MALDI–FTICR–MS) studies have resolved the in situ distributions of low-molecular-weight lignin aromatic derivatives in brown rot fungal decayed wood^24^. Despite using a 15-T FTICR mass spectrometer with an ultra-high-mass resolving power and mass accuracy, confident molecular annotation remains a notable challenge in this stand-alone method. Therefore, previous studies used different orthogonal techniques for more confident molecular annotations. For example, MALDI–MSI along with a tandem MS (MS/MS) approach enabled scientists to study the chemistry of underlying microbial interactions among cuticular bacteria and pathogenic fungi associated with fungus-growing ants^21^. Additionally, a multimodal imaging approach using MALDI–MSI and liquid extraction surface analysis–MS/MS (LESA–MS/MS) enabled scientists to confidently annotate and map a broad range of metabolites involved in a tripartite symbiosis system of moss, cyanobacteria and fungus^26^. However, these techniques have been applied to cultured systems where the molecular signals are more enriched and homogeneous than naturally evolved systems.

Another challenge of MALDI–MSI is comprehensive proteome imaging, as MALDI ions have a low-charge state (primarily singly charged ions), putting protein ions out of the mass range of FTICR instruments. Although protein coverage can be improved with on-tissue digestion, in situ MS/MS peptide identification remains challenging due to low signal-to-noise ratios and high spectral complexity that impede database identifications^27^.

**Fig. 1:**
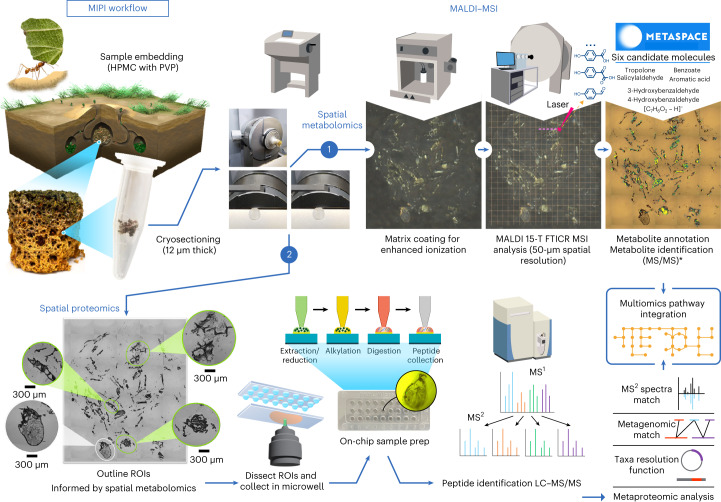
**MIPI.** Schematic workflow of the MIPI approach that combines two complementary microscale spatial modalities, (1) MALDI–FTICR–MSI and (2) microPOTS; HPMC, hydroxypropyl methylcellulose; PVP, polyvinylpyrrolidone. *Orthogonal MS/MS (MS2) analyses confirmed the metabolite identifications mapped by MALDI MS1 images.

To overcome these challenges, we establish a systematic workflow by (1) integrating two spatial techniques to improve molecular annotations, (2) applying a low-volume sample-processing strategy to enhance protein detection and (3) implementing complementary MS/MS techniques to cross-validate the detected functions. Our workflow is based on a metabolome-informed proteome imaging (MIPI) approach (Fig. 1). In addition to MALDI–FTICR–MSI, we use our microdroplet processing in one pot for trace samples (microPOTS) approach for complementary proteomic profiling of metabolome-informed regions of interest (ROIs)^28^. The microPOTS approach uses a low-volume sample-processing strategy and low-flow separation coupled to sensitive MS/MS instrumentation. Although existing sample-processing platforms for low-input proteomics use different approaches^29–31^, a common goal is to advance miniaturization strategies for sample preparation to minimize sample loss and maximize digestion kinetics^32^. Our workflow provides quantitative proteomic measurements combined with submillimeter spatial context for untargeted metabolome mapping of complex and heterogeneous biological tissues.

**Fig. 2:**
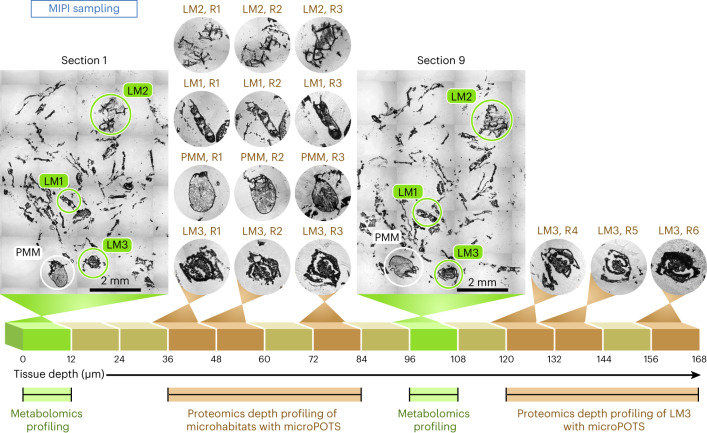
MIPI depth profiling of microhabitat zones. An illustration of our MIPI depth profiling through microhabitats (LM1, LM2, PMM and LM3) with replicate sections (R1, R2, R3, R4, R5 and R6).

In this study, we establish and implement our MIPI workflow for a comprehensive molecular characterization of unique microhabitats within a heterogeneous and multimember lignocellulose-degrading consortium. First, MALDI–MSI analyses generate systematic images of 12-μm-thick sections through the embedded fungal garden sample (Fig. 1, workflow 1). These metabolite images allow us to visualize dynamic molecular networks across distinct sample regions and pinpoint microhabitats of interest for subsequent metaproteomic imaging in adjacent sections. Second, our microPOTS metaproteomic imaging approach profiles enzymes in these microscale habitats of interest to link molecular signatures with species-specific enzymes from each zone (Fig. 1, workflow 2). Analysis of replicate sections with our MIPI workflow enables multiple measurements and depth profiling of microhabitat zones (Fig. 2 and Statistics and reproducibility).

**Fig. 3:**
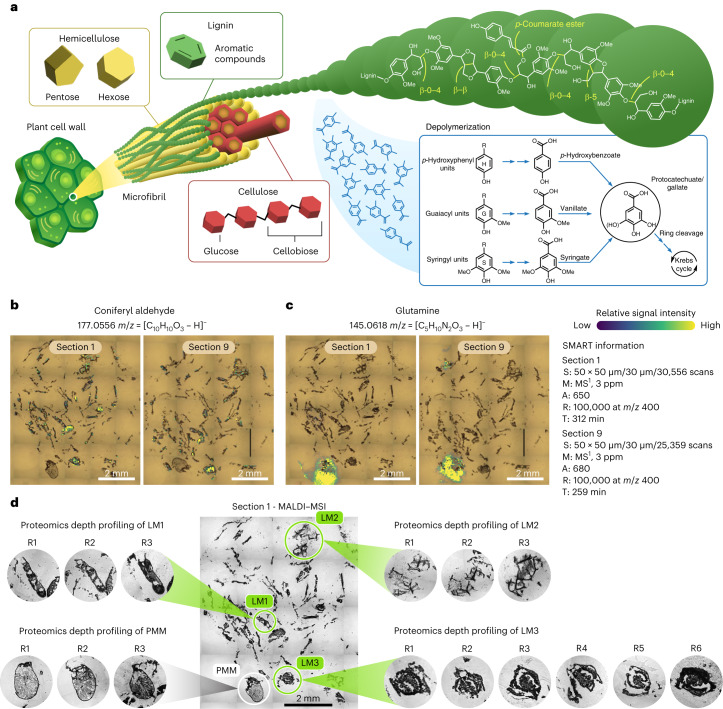
Identifying microhabitats in the fungal garden ecosystem with MALDI metabolite imaging. **a**, Molecular structure of lignin. **b**,**c**, Overlaid ion image obtained by MALDI–MSI and optical images of 12-μm-thick fungal garden sections with distinct microscopic features. SMART^52^ information of the MSI data for sections 1 and 9: S, step size/spot size/total scans; M, molecular confidence; A, annotations (METASPACE, KEGG, ≤20% FDR, [M – H]^–^[M + Cl]^–^); R, resolving power; T, time of acquisition. **b**, LM example ion image of coniferyl aldehyde representing the spatial pattern of lignin-related secondary metabolites. **c**, PMM example ion image of glutamine demonstrating a spatial pattern characterized mainly by primary metabolites and localized only in the ant-related feature. **d**, Regions selected for complementary spatial proteomics analysis. ROIs denoted in green in section 1 represent three LMs (LM1, LM2 and LM3) mapped as possible lignocellulose degradation hot spots, and the ROI denoted in white is a PMM mapped as a possible ant metabolism zone. Metabolome-guided ROIs were collected from adjacent section replicates, LM1, LM2 and PMM, from three replicate sections (R1–R3) and LM3 from six replicate sections (R1–R6) for subsequent metaproteomic imaging.

Integration of the data from our multimodal MS approaches provides crucial information on underlining molecular mechanisms in each distinct microhabitat. Here, we link taxon-specific enzymes to lignocellulose (that is, hemicellulose, cellulose and lignin; Fig. 3a) deconstruction and visualize lignin degradation at the pathway level. Application of our MIPI platform to capture taxon-specific metabolic activity in micrometer-scale habitats clearly demonstrates the potential of MIPI to map metabolic differences across diverse heterogeneous samples with pathway-level resolution.

## Results

### Low-molecular-weight lignin products at a micrometer scale

Untargeted MALDI–FTICR–MSI analysis revealed heterogeneous spatial distributions of various molecular features across the fungal garden sections. We leveraged the METASPACE annotation platform to search against the Kyoto Encyclopedia of Genes and Genomes (KEGG) database and tentatively annotated 364 and 299 unique features (Supplementary Table 1a,b) that colocalized metabolomic signatures with distinct microhabitats detected across sections 1 (0–12 µm) and 9 (96–108 µm; Fig. 2), respectively. The MALDI images mapped the presence of phenylpropanoids, benzaldehydes, flavonoids, plant hormones, Krebs cycle compounds, sugars, amino acids and other molecules that were produced by the complex fungal garden community.

Within our data, we focused on two predominant and distinct spatial patterns that colocalized with morphologically unique micrometer-scale habitats in the garden ecosystem. We named these ROIs lignin microhabitat (LM) and primary metabolite microhabitat (PMM). As depicted in Fig. 3b,c, the two MALDI image replicates reproducibly detected the same spatial arrangement of the LM and PMM, although collected approximately 100 µm apart. The LM contained an accumulation of low-molecular-weight lignin products, such as phenylpropanoids (the ions matched the molecular formulas of coniferyl alcohol, coniferyl aldehyde, sinapoyl aldehyde and 4-coumaryl alcohol), their corresponding acids (the ions matched the molecular formulas of coumarate, cinnamate, ferulate, vanillate, gallate and caffeate) and benzaldehydes (the ions matched the molecular formulas of veratraldehyde and 4-hydroxyacetophenone; Fig. 3a,b). The PMM contained soluble sugars, amino acids and fatty acids, by molecular formula annotations, which mapped to a single ROI that seemed to represent an ant egg-like feature (Fig. 3c). METASPACE metabolite identifications leveraged our high-mass accuracy high-resolution MSI datasets to perform MS^1^-based metabolite annotation against the KEGG database. MS^2^-based metabolite identifications confirmed the identity of our metabolites in pathways of interest (see Methods).

Informed by the metabolome-specific features, three distinct LM ROIs and one PMM ROI (denoted in Fig. 3d in green (LM1, LM2 and LM3) and white (PMM), respectively) were further investigated at the proteome level. The LM1, LM2 and LM3 ROIs were mapped as lignin degradation hot spots; hence, they were dissected and analyzed in our subsequent spatial proteomics approach to obtain comprehensive enzymatic contributions of fungi to lignocellulose degradation. Additionally, the PMM was profiled at the protein level to capture the taxonomic and functional assignments and characterize this likely ant-related feature.

### Proteome depth analyses in metabolome-informed microhabitats

Sections in close proximity to each of the two MALDI-imaged (Figs. 2 and 3b,c) fungal garden sections retained the same spatial arrangement of the microhabitat features. These sections, therefore, were considered replicates of each other (Fig. 3d, R1–R3) and were suspected to contain enzymes that catalyzed metabolic reactions in the targeted microhabitats (that is, LM and PMM). As LM3 was a larger microhabitat along the depth gradient, six sections corresponding to LM3 were collected in contrast to three sections for the other three microhabitats (that is, LM1, LM2 and PMM) to demonstrate the reproducibility in metaproteomic data. Each of the four microhabitats was isolated from the collected sections via laser-capture microdissection and was independently processed by microPOTS, where the resulting peptides were analyzed by liquid chromatography–MS/MS (LC–MS/MS). Acquired MS/MS data were searched against our customized protein database following taxonomic and functional annotations using metaproteomic workflows (Supplementary Fig. 1).

To annotate the LC–MS/MS data, we compiled a total of 147 leaf-cutter ant fungal garden metagenomes and genomes of known resident microbes together with the proteins predicted using the de novo metaproteomics method^33^. Our reference database contains 50 million proteins of known members in the consortium that are grouped into 24 million clusters based on sequence similarity to annotate the high-resolution MS/MS spectra with stringent matching criteria. A total of 7,392 unique and taxon-specific peptides attributed to 2,339 annotated protein clusters were identified from the four microhabitat ROIs. As a result, 93% of the protein clusters were assigned to 1,105 KEGG Ortholog (KO) and 195 KEGG metabolic pathways (Supplementary Table 2).

**Fig. 4:**
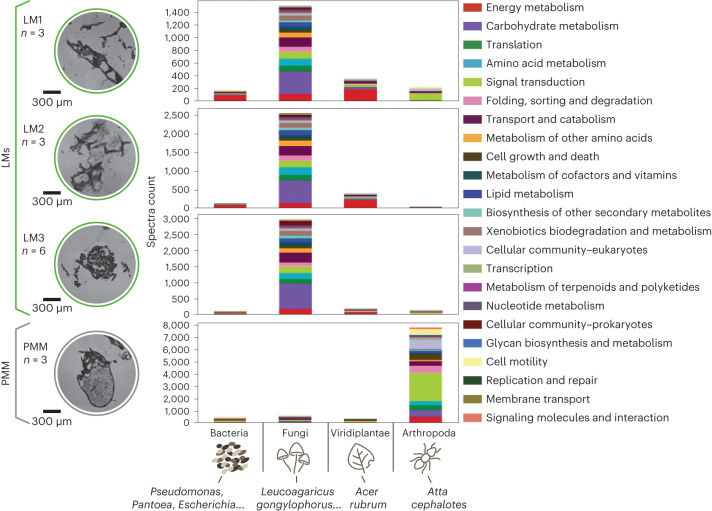
Metaproteomic-informed taxonomic and functional profiles in the microscale regions of interest from the fungal garden ecosystem. The bar graph includes redundant peptides (listed in Supplementary Table 4) with conserved sequence homology across organisms; n, number of section replicates.

Metaproteome analysis of PMMs provides in-depth taxonomic and functional characterizations in regions with ant-related features. We applied three complementary protein- and contig-based methods to make conservative taxonomic assignments (Supplementary Fig. 1). A total of 5,178 unique peptides were assigned the to phylum Arthropoda (the phylum of *Atta cephalotes*) and localized predominantly in the PMM ROI (Supplementary Table 2). This result supports our hypothesis of the presence of an ant egg in this region based on the morphological features. Functional annotation from KEGG provided insights into biological processes localized at the PMM ROI. About 29% of Arthropoda-associated peptide–spectra matches (PSMs) were mapped to pathways related to signal transduction (Fig. 4). Detection of the Arthropoda membrane protein flotillin (K07192), sorbin and SH3 domain-containing protein 1 (K06086) mapped the part of the insulin signaling pathway (ko04910) related to glucose uptake. Additionally, the detection of ribosomal protein S6 kinase-β (K04688, EC 2.7.11.1) and small subunit ribosomal protein S6e (K02991) mapped the part of the insulin signaling pathway leading to protein synthesis. We also detected 18 and 23 Arthropoda-specific proteins related to the metabolic pathways focal adhesion (ko04510) and biosynthesis of amino acids (ko01230), respectively (Supplementary Table 3a,b). The detection of Arthropoda-specific proteins supported our hypothesis that there was an ant egg in the PMM. Functional analysis of the metaproteomic data with this pathway-level resolution further suggested that ant eggs contain nutrient-rich fluids with high levels of amino acids and proteins to support larva development.

Metaproteome analysis of the metabolome-informed LMs revealed enzymatic contributions of fungi to lignocellulose degradation. The qualified peptides with at least two PSMs and the cluster proteins with at least two qualified peptides were used for taxonomic and functional profiling of the ROIs (Fig. 4 and Supplementary Table 4). Consistent with the MALDI–MSI results, the targeted microhabitats were enriched with proteins that primarily contribute to pathways related to lignocellulose degradation (for example, carbohydrate metabolism, transport and catabolism and signal transduction). We further extracted unique PSMs (unique to a single protein sequence in the multimember database) to conservatively estimate the taxonomic contributions to the detected functions. In total, 1,825 unique peptides detected in LM regions were identified as specific to fungi, in contrast to the 47 and 552 peptides that were unique to the coexisting bacterial^6^ and Viridiplantae (green plants) members, respectively (Supplementary Table 2). This result implies the predominant role of fungi in lignocellulose degradation within the targeted microhabitats. The detected bacterial proteins participate mainly in energy metabolism pathways that enable the resident bacteria to transform previously digested carbohydrates into molecules and thus benefit the bacteria and consortium^6^.

### Lignocellulose degradation pathways in unique microhabitats

MIPI analyses provide pathway-level information about the role of fungi in the garden ecosystem that leads to lignocellulose degradation and ant nutrient provisioning. Specifically, our metaproteomic data revealed a plethora of fungus-specific enzymes involved in the metabolism of carbohydrates by microPOTS metaproteomes (Table 1 and Supplementary Table 5). Low-molecular-weight lignin metabolites profiled in our MALDI–MSI data correlated with our microPOTS data, which identified a lignin-degrading auxiliary enzyme glyoxal/methylglyoxal oxidase (K20929, EC 1.2.3.15). Hemicellulose degradation requires a diverse set of fungal enzymes not only because of hemicellulose complexity but also because of its connection with the other plant cell wall polysaccharides^34^. Endo-1,4-β-xylanase (K01181, EC 3.2.1.8), one of the key enzymes responsible for breaking down β‐1,4‐xylan chains into xylo‐oligosides, was detected in our microPOTS data, as well as α-l-arabinofuranosidase (K01209, EC 3.2.1.55) and exo-1,4-β-xylosidase (K15920, EC 3.2.1.37). The major mannan-degrading enzymes that ultimately release d-mannose exo-β-d-mannanase (K01192, EC 3.2.1.25) and endo-1,4-β-mannanase (K19355, EC 3.2.1.78), which deconstruct the β-1,4-mannan backbone, and the auxiliary enzymes α-galactosidase (K07407, EC 3.2.1.22) and β-glucosidase (K05349, EC 3.2.1.21) were also detected. A starch-degrading enzyme, glucoamylase (K01178 EC 3.2.1.3), was identified along with several pectinolytic enzymes that belong to hydrolase, lyase and esterase families, which act on the pectic substrate through reactions of depolymerization and de-esterification. Across the distinct ROIs, the presence of pectinesterase (K01051, EC 3.1.1.11), pectate lyase (K01728, EC 4.2.2.2) and rhamnogalacturonan endolyase (K18195, EC 4.2.2.23) as well as hydrolases such as galacturan 1,4-α-galacturonidase (K01213, EC 3.2.1.67) and rhamnogalacturonan acetylesterase (K15530, EC 3.1.1.86) were observed. d-Galacturonate reductase (K18106, EC 1.1.1.-) and its substrate galacturonic acid were profiled using our multiomics MIPI workflow and showed high spatial colocalization and correlation in the imaged ROIs across two modalities. Further, our microPOTS data revealed the presence of cellulases (enzymes that degrade the most abundant polysaccharide of the plant cell wall). Specifically, endoglucanase (K01179, EC 3.2.1.4) and cellulose 1,4-β-cellobiosidase (K01225, EC 3.2.1.91) with the previously mentioned β-glucosidase (K05349, EC 3.2.1.21) ultimately decompose cellulose into units of glucose.

**Table 1:** List of detected enzymes involved in lignocellulose biomass degradation with their taxonomic assignments and ROI-specific localization

Substrate specificity	KO number	EC number	Enzyme	Taxonomic annotation(s)	LM1 (*n* = 3)	LM2 (*n* = 3)	LM3 (*n* = 6)	PMM (*n* = 3)
Lignin	K20929	1.2.3.15	Glyoxal/methylglyoxal oxidase	Fungi	**O**	**O**	**O**	X
Hemicellulose	K01181	3.2.1.8	Endo-1,4-β-xylanase	Fungi/*Leucoagaricus*	**O**	X	**O**	X
Hemicellulose	K01209	3.2.1.55	α-l-Arabinofuranosidase	Fungi/*Leucoagaricus*	**O**	**O**	**O**	**O**
Hemicellulose	K15920	3.2.1.37	Exo-1,4-β-xylosidase	Fungi/*Leucoagaricus*	**O**	**O**	**O**	X
Hemicellulose	K01192	3.2.1.25	Exo-β-d-mannanase	Fungi	X	**O**	**O**	X
Hemicellulose	K19355	3.2.1.78	Endo-1,4-β-mannanase	Fungi	X	**O**	X	X
Hemicellulose	K07407	3.2.1.22	α-Galactosidase	*Leucoagaricus*	X	**O**	**O**	X
Hemicellulose/cellulose	K05349	3.2.1.21	β-Glucosidase	Fungi/*Leucoagaricus*	**O**	**O**	**O**	X
Starch	K01178	3.2.1.3	Glucoamylase	Fungi/*Leucoagaricus*	**O**	**O**	**O**	X
Pectin	K01051	3.1.1.11	Pectinesterase	Fungi/*Leucoagaricus*	**O**	**O**	**O**	X
Pectin	K01728	4.2.2.2	Pectate lyase	Fungi/*Leucoagaricus*	**O**	**O**	**O**	**O**
Pectin	K18195	4.2.2.23	Rhamnogalacturonan endolyase	Fungi/*Leucoagaricus*	X	**O**	**O**	X
Pectin	K01213	3.2.1.67	Galacturan 1,4-α-galacturonidase	Fungi	X	**O**	**O**	X
Pectin	K15530	3.1.1.86	Rhamnogalacturonan acetylesterase	Fungi/*Leucoagaricus*	**O**	**O**	**O**	X
Pectin	K18106	1.1.1.-	d-Galacturonate reductase	Fungi/*Leucoagaricus*	**O**	**O**	**O**	X
Cellulose	K01179	3.2.1.4	Endoglucanase	Fungi/*Leucoagaricus*	**O**	**O**	**O**	X
Cellulose	K01225	3.2.1.91	Cellulose 1,4-β-cellobiosidase	Fungi/*Leucoagaricus*	**O**	**O**	**O**	X
Chitin	K01183	3.2.1.14	Chitinase	Fungi/Arthropoda^a^	**O**	**O**	**O**	**O**

A full list of peptides assigned to the cluster and corresponding KO numbers along with their functional annotation, taxonomic assignments and ROI-specific localization have been included in Supplementary Table 5. A peptide was considered present if it was detected in at least two section replicates within each ROI. The cluster was identified with at least two peptides detected within the ROI that previously met peptide identification criteria.

**O**, detected; X, absent or below detection limits.

^a^The chitinase related to the phylum Arthropoda was localized in the PMM ROI.

Different from lignocellulose degradation, chitin was suspected to be metabolized by more diverse members of the garden ecosystem. The chitinase (K01183, EC 3.2.1.14) was found to have diverse taxonomic assignments (Arthropoda and fungi) between the four ROIs. Detected peptides assigned to this chitin-degrading hydrolytic enzyme belonged to Arthropoda in the PMM ROI and to fungi in ROIs LM1, LM2 and LM3. We acknowledge that the phylogenetically relevant species share sequence homology. Therefore, the detected proteins were assigned to higher taxonomic ranks using three conservative annotation methods (Supplementary Fig. 1).

### MIPI spatial multiomics details lignin degradation pathways

Integrative multiomics analyses at the spatial level can provide an informative view of active biological pathways at microscale resolution. To further resolve the metabolic pathways of lignocellulose degradation that were primarily mediated by fungi in the distinct micrometer-scale hot spots, we integrated the results of the spatial multiomics implemented in MIPI. Integrated metabolome and proteome data generated using our MIPI approach can rapidly identify even subtle fluctuations of microbial activities within heterogeneous microhabitats. The reconstruction of spatial microbial activities was achieved by mapping the detected metabolites and the paired enzymes to the respective KEGG metabolic pathways. Focusing on catabolic pathways of small lignin-derived compounds (that is, aromatic degradation and ring cleavage pathways) provided insights into fungal intracellular metabolism of lignin deconstruction products^35^.

**Fig. 5:**
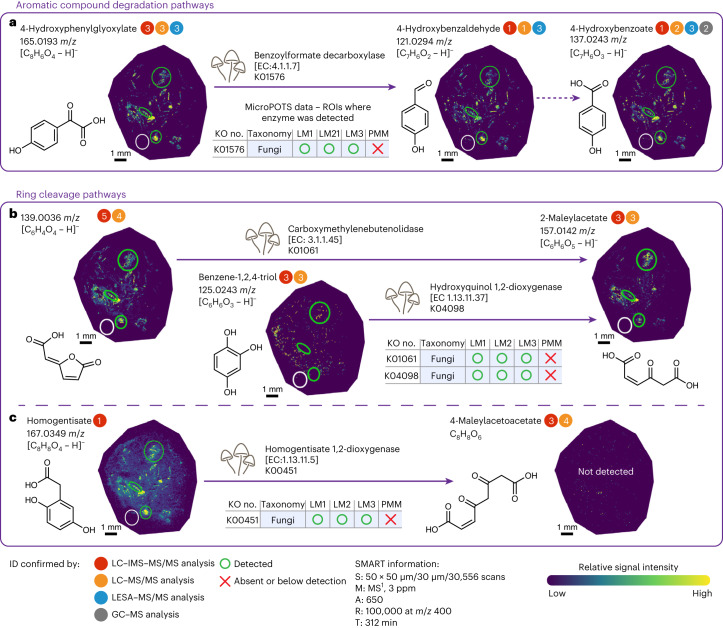
Spatial multiomics integration unravels complex catabolic pathways in the leaf-cutter ant fungal garden ecosystem. The following are the included pathways. **a**, Conversion of 4-hydroxyphenylglyoxylate by fungal benzoylformate decarboxylase. **b**, Two pathways of aromatic ring cleavage by fungi to 2-maleylacetate. **c**, Homogentisic pathway of homogentisate ring cleavage by fungi. Metabolite MALDI–FTICR–MSI images visualize the relative abundance of each metabolite across section 1. Metabolite ion images of replicate section 9 are provided in Supplementary Fig. 3. The three green circles and one white circle depict the LM and PMM ROIs, respectively (LM1, *n* = 3; LM2, *n* = 3; LM3, *n* = 6; PMM, *n* = 3). Complementary microPOTS enzyme identifications are shown above each reaction arrow, with the table denoting if the enzyme was detected (O) or absent (X) in LM1, LM2, LM3 or PMM. An enzyme was considered present if it was detected in at least two section replicates within each ROI. A detailed list of enzymes with their corresponding peptides, taxonomic assignments and ROI-specific localization is provided in Supplementary Table 10. Colored circles next to the metabolite names indicate that metabolite identities were confirmed by different orthogonal techniques via MS/MS (MS^2^ or MS/MS), included in Supplementary Fig. 2. Numbers within each circle indicate metabolite identification metrics (that is, 1 (high) to 5 (low))^36^, as detailed in Supplementary Fig. 2. The molecular formula [C_6_H_4_O_4_ – H]^–^ matches to candidate molecules 4-carboxymethylenebut-2-*en*-4-olide (the substrate of carboxymethylenebutenolidase → 2-maleylacetate) and 5-formyl-2-furoate in the KEGG database.

**Fig. 6:**
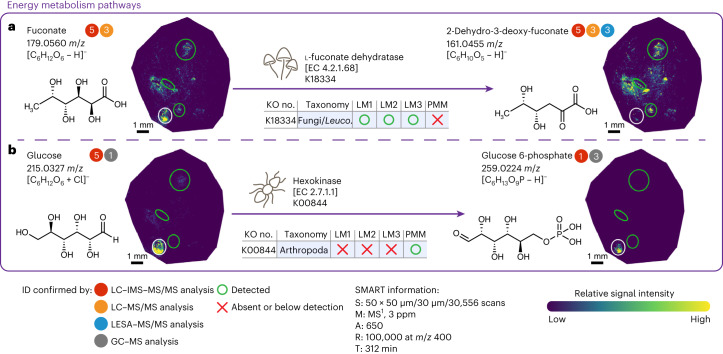
Spatial multiomics integration resolves mass isomers and unravels taxon-specific pathways in the leaf-cutter ant fungal garden ecosystem. The following are the included taxon-specific pathways. **a**, Catabolic pathway of fuconate by fungi. **b**, Phosphorylation reaction of fuconate’s mass isomer glucose by the enzyme hexokinase, taxonomically assigned to the phylum Arthropoda. Metabolite MALDI–FTICR–MSI images visualize the relative abundance of each metabolite across section 1. Metabolite ion images of replicate section 9 are provided in Supplementary Fig. 3. The three green circles and one white circle depict the LM and PMM ROIs, respectively (LM1, *n* = 3; LM2, *n* = 3; LM3, *n* = 6; PMM, *n* = 3). Complementary microPOTS enzyme identifications are shown above each reaction arrow, with the table denoting if the enzyme was detected (O) or absent (X) in LM1, LM2, LM3 or PMM. An enzyme was considered present if it was detected in at least two section replicates within each ROI. A detailed list of enzymes with their corresponding peptides, taxonomic assignments and ROI-specific localization is included in Supplementary Table 10. Colored circles next to the metabolite names indicate that metabolite identities were confirmed by different orthogonal techniques via MS/MS (MS^2^ or MS/MS), included in Supplementary Fig. 2. Numbers within each circle indicate metabolite identification metrics (that is, 1 (high) to 5 (low))^36^, as detailed in Supplementary Fig. 2.

The presence of metabolites from the metabolic pathways was confirmed by using orthogonal MS/MS-based techniques on extracts from bulk fungal garden samples. Using multiple techniques provided complementary MS^2^-based metabolite identifications (Supplementary Fig. 2) with identification confidence metrics^36^ (level 1 (high) to level 5 (low); Figs. 5 and 6 and Supplementary Fig. 3; confidence numbers are shown in each colored circle). These techniques included LC–ion mobility spectrometry–MS/MS (LC–IMS–MS/MS) using hydrophilic interaction LC (HILIC) separations and data-independent acquisition (DIA;^37^ Figs. 5 and 6, red circles), LC–MS/MS using HILIC and reversed-phase (RP) LC separations and data-dependent acquisition (Figs. 5 and 6, orange circles) and gas chromatography–MS (GC–MS; Figs. 5 and 6, gray circles). In addition, LESA–MS/MS provided spatially resolved metabolite identifications from a fungal garden section (Figs. 5 and 6, blue circles). For method details, see MS^2^-based metabolite identification, and for metabolite identification confirmation results, see Supplementary Tables 6–9 and 14 and Supplementary Figs. 2–6.

Our integrated MIPI data mapped an aromatic compound degradation pathway leveraging the fungal enzyme benzoylformate decarboxylase (K01576, EC 4.1.1.7). We detected benzoylformate decarboxylase, a marker of aromatic compound degradation where 4-hydroxyphenylglyoxylate is converted to 4-hydroxybenzaldehyde (Fig. 5a and Supplementary Fig. 3), which can be subsequently transformed to 4-hydroxybenzoate. The presence of metabolites from this pathway was confirmed by LC–IMS–MS/MS, LC–MS/MS, LESA–MS/MS and GC–MS. Integrated metabolomic and metaproteomic evidence showed a high correlation at the spatial scale between the peptide APSIEPGALSPDVTR associated with the enzyme benzoylformate decarboxylase and the detected substrate (correlation value > 0.999; adjusted *P* value of 8.27 × 10^−17^) and product (correlation value > 0.999; adjusted *P* value of 3.61 × 10^−38^) along the pathway (Supplementary Table 14).

Our MIPI data discovered diverse aromatic ring cleavage pathways. Two distinct fungal ring cleavage pathways yielded an accumulation of the same product, 2-maleylacetate (Fig. 5b). The first suggested pathway of ring cleavage through carboxymethylenebutenolidase (K01061, EC 3.1.1.45) enzymatic processing showed high substrate–enzyme–product spatial correlation (the correlation values between the peptide IIDEFIFR and the enzyme’s substrate and products were 0.956 and 0.993 with adjusted *P* values of 1.22 × 10^−6^ and 9.07 × 10^−11^, respectively). The accurate molecular formula for the substrate ([C_6_H_4_O_4_ – H]^–^) matched that of two compounds within the KEGG database, 4-carboxymethylenebut-2-*en*-4-olide and 5-formyl-2-furoate. Carboxymethylenebutenolidase can convert 4-carboxymethylenebut-2-*en*-4-olide into 2-maleylacetate.

Another conversion of benzene-1,2,4-triol (hydroxyquinol) to 2-maleylacetate by hydroxyquinol 1,2-dioxygenase (K04098, EC 1.13.11.37) showed high spatial correlation between the peptide NGLLPPFPR ascribed to the enzyme and product (correlation value of 0.988; *P* value of 2.35 × 10^−9^). Benzene-1,2,4-triol, the substrate of the proposed reaction, was likely almost entirely converted to the final product during the snapshot in time when the sample was collected. Although benzene-1,2,4-triol was detected in relatively low amounts via MALDI–MSI, its presence in a bulk fungal garden metabolite extract was confirmed by LC–IMS–MS/MS and LC–MS/MS.

Another ring cleavage pathway was the homogentisate central pathway (a central pathway for catabolism of phenylalanine and tyrosine), which was indicated by the detection of the fungal enzyme homogentisate 1,2-dioxygenase (K00451, EC 1.13.11.5), and its corresponding substrate homogentisate (Fig. 5c) showed a high spatial correlation between the peptide LPLDSDKIDFVEGLK and substrate (correlation value of 0.965; *P* value of 3.93 × 10^−7^). The presence of homogentisate was confirmed by bulk metabolomics analyses using a standard and our LC–IMS–MS/MS DIA workflow^37^ that distinguished it from mass isomers. Although the product resulting from aromatic cleavage of homogentisate (4-maleylacetoacetate) was below the detection limits of the MALDI–FTICR analysis, its presence was confirmed by LC–MS/MS and LC–IMS–MS/MS via analyses of bulk fungal garden extract.

Our integrated MIPI data resolved colocalized/coeluted metabolite mass isomers, enabling us to map additional underlying taxon-specific energy metabolism pathways. A part of the non-phosphorylated 6-deoxyhexose sugar metabolic pathway, in which the fungal enzyme l-fuconate dehydratase (K18334, EC 4.2.1.68) converts l-fuconate to 2-dehydro-3-deoxy-l-fuconate, is depicted in Fig. 6a. Molecular annotations of fuconate and 2-dehydro-3-deoxy-fuconate were confirmed in bulk garden extracts via LC–MS/MS and LC–IMS–MS. Molecular annotation of 2-dehydro-3-deoxy-fuconate was also confirmed in a garden section via LESA–MS/MS. Although we were expecting metabolites and their corresponding enzymes to colocalize within each ROI, the enzyme l-fuconate dehydratase did not adhere to the expected localization pattern. The enzyme was detected in LM1–LM3 and was not detected in the PMM. However, the corresponding substrate was detected in all ROIs (annotated using a less stringent criterion false discovery rate (FDR) ≤ 50%; Supplementary Fig. 4), indicating that there was a mass isomer present in the PMM. Leveraging the ultra-high resolving power of the MALDI–FTICR technique, we were able to distinguish the mass isomers in negative ion mode based on ionization specificity because fuconate readily ionizes by releasing the proton (C_6_H_12_O_6_ – H^−^; Fig. 6a), and its mass isomer glucose favors attaching a chloride ion (C_6_H_12_O_6_ + Cl^−^; Fig. 6b). Our complementary metaproteomic analyses confirmed the existence of glucose metabolism in the PMM ROI by revealing the hexokinase enzyme (K00844, EC 2.7.1.1) that was taxonomically assigned to the phylum Arthropoda (Fig. 6b). Integration of the results revealed high enzyme–product correlation (correlation value of 0.992; *P* value of 2.07 × 10^−10^). Glucose conversion to glucose-6-phosphate was further confirmed using GC–MS and LC–IMS–MS/MS analyses on bulk fungal garden samples.

## Discussion

Metabolomics pathways in the fungal gardens of leaf-cutter ants remain highly elusive and unexplained due to complex garden community properties, including the wide diversity of plants foraged by the ants, taxonomic diversity of community members, highly dynamic molecular changes across a vertical gradient of biomass degradation, environmental changes and the corresponding impact on the garden microbiota, pathogenic properties of some consortia and so on. Hence, the key contributors and molecular mechanisms of in situ lignocellulose degradation remain unknown. Previous publications of plant biomass decomposition pathways in this fungal garden ecosystem have leveraged single-omics analyses, which only reflect a piece of the biochemical processes (that is, metabolite or enzyme)^7,8^. In addition, the molecular signals are often diluted in samples that are collected in bulk. To fill this critical knowledge gap and overcome the technical challenges, we established our MIPI workflow by integrating data from two spatial multiomics MS modalities (MALDI–MSI and microPOTS) to identify metabolic hot spots and resolve the functional pathways (Fig. 1). MALDI–MSI pinpointed habitats of lignin degradation with microscale accuracy. Metaproteomic depth profiling through these microhabitats then unveiled region-specific enzymes with ultrasensitive microPOTS processing. These results highlight MIPI’s ability to perform deep multiomics uniquely targeted to hot spots of metabolic activity.

Integrated multiomic data collected from metabolic hot spots via a MIPI workflow revealed taxon-specific metabolomic networks and mechanistic pathways. Fungi were identified as the main drivers of lignin degradation in the studied system. Our microscale metabolomics analysis revealed the presence and colocalization of numerous secondary metabolites with differential abundance patterns across fungal garden sections, including the detection of veratryl aldehyde, which was previously reported as a possible product of hydrogen peroxide-dependent oxidation of veratryl alcohol that acts as a cofactor of white rot fungus lignin peroxidase^38,39^. Among various fungal carbohydrate-active enzymes, our metaproteomics data showed a high abundance of one fungal ligninolytic glyoxal/methylglyoxal oxidase enzyme, which is known to produce hydrogen peroxide to support lignin degradation by peroxidases^40^. Our integrated MIPI data mapped catabolism pathways with one caveat, the reconstructed intracellular microbial aromatic pathways are not exclusively related to lignin catabolism because aromatics can be derived from sources other than lignin. We unveiled part of aromatic compound degradation pathways by mapping benzoylformate decarboxylase with its substrate 4-hydroxyphenylglyoxylate. It has been previously reported that mandelate and 4-hydroxymandelate can be metabolized by filamentous fungi^41^, thus the detected substrates have the potential to be products of mandelate pathway conversion. Notably, there are indications that 4-hydroxyphenylglyoxylate can be a product of lignin degradation via oxidation of an aryl C2 keto-aldehyde precursor, converted from phenylcoumaran and diarylpropane units in lignin^42^. In addition, detection of fungal ring cleavage dioxygenases reconstructed the critical ring-opening step in the catabolism of aromatic compounds, leading to the degradation of homogentisate to the products that enter the Krebs cycle. Leveraging an orthogonal technique, we confirmed the presence of homogentisate by LC–IMS–MS/MS and distinguished it from its mass isomers vanillate and 4-hydroxymandelate (Supplementary Fig. 5). The co-detection of cofactors, enzymatic proteins and metabolic products of the same pathway across multiple sections provided more molecular evidence for fungal contributions to lignin degradation. Abundant fungus-specific carbohydrate-active enzymes with specificity to hemicellulose, cellulose, pectin and amylose (Supplementary Table 13a,b) suggest that fungi are the dominant drivers of plant cell wall degradation in the fungal garden ecosystem.

Our MIPI analysis provided molecular evidence that the unknown PMM microscopic feature was an *A. cephalotes* egg. Our metabolomics characterization showed a plethora of primary metabolites in the putative egg, and our spatial metaproteomics profiling showed a plethora of enzymes, mainly related to signal transduction. Mapping these detected enzymes into the insulin signaling pathway aligned with previous knowledge that *A. cephalotes* has all of the core genes of a highly conserved insulin signaling pathway that plays a key role in many processes, including reproduction, metabolism, growth and aging^43,44^. Leveraging our MIPI capability to spatially profile a plethora of metabolites and peptides provided molecular insights into taxon-specific activities in this multimember heterogeneous ecosystem. Mechanistic understanding of how this natural ecosystem has evolved over 8–12 million years^45^ to efficiently degrade plant biomass can aid in the biological production of biofuel precursors and bioproducts from plant biomass. The MIPI measurements provided a comprehensive view of underlying biological pathways, empowered more effective investigations and augmented our understanding of the existing interactions between the members within the micrometer-scale environment. We demonstrated the ability of this MS micrometer-scale multiomic workflow to understand microhabitats at the pathway level. Our MIPI approach can be applied to numerous complex and heterogeneous biological systems ranging from other complex consortia to unique tissue microenvironments within cellular systems. In this study, our ROIs in the fungal garden ecosystem were on the microscale and ranged from 400,000 to 700,000 µm^2^ in area and 12 µm in depth. But, as depicted by our optical images in Fig. 3d, the biological material in each ROI was sparse; on average, each region only contained ≤50% tissue. We envision that MIPI can evolve to explore the biomolecular signatures present in tissues at higher resolution, even cell resolution, with nanodroplet processing for spatial proteomics^27^. In this study, we did not use each pixel of our MALDI imaging metabolite data. Instead, we used the average pixel intensity for each ROI because that corresponded to the proteomic data level of the sample region. Improving the proteomic spatial resolution will enable future studies to navigate all the data and learn everything about each pixel in the data and how they co-depend. In addition, increasing the spatial resolution along with on-tissue chemical derivatization^46^ will enable the detection of minor components and their metabolic dynamics. These future developments will allow us to define the spatial metabolome of complex microbial systems with enhanced accuracy and precision. Depending on sample complexity and the spatial resolution needed for biological pathway analysis, the MIPI workflow can be expanded to interrogate metabolome-guided ROIs that range from tissue functional units to a single cell.

The accurate mass and isotopic pattern analyses of our MALDI–FTICR images narrow down the metabolite identity of each *m*/*z*; yet they do not provide definitive information about the specific chemical structure. Additional information, such as fragmentation patterns and comparisons with databases and reference compounds, is required to confidently assign^36^ the chemical structure of a metabolite. Compared to other omics approaches, metabolomics presents unique challenges due to the vast chemical complexity of known metabolites found in nature^47^. Unlike nucleotides or amino acids that make up DNA, RNA and protein, metabolites do not consist of repeated structural elements and can vary in structure, size, polarity and concentration. Due to these challenges, we used diverse MS instruments (that is, LC–MS/MS and LESA–MS/MS (Q Exactive Plus Orbitrap), GC–MS (single quadrupole MSD 5975C) and LC–IMS–MS/MS (6560 Drift Tube Ion Mobility Q-TOF)) to provide high-quality spectral data. Even so, we obtained different levels of metabolite identification confidence (Figs. 5 and 6 and Supplementary Fig. 2). For the highest level of confidence (level 1: confirmed structure), we analyzed pure standards with the same method and instrument to compare MS^1^, MS/MS fragments and retention time (RT) to our fungal garden metabolite. Yet, standards were not available for all metabolites because their synthesis can be expensive and time consuming. For confidence level 2 and 3 identifications, we matched MS^1^ and MS/MS fragments to in-house libraries and the literature. By using diverse analyses, most of the metabolites were characterized multiple times where at least one analysis contained a confidence level of 3 or better. A minority of analyses had poor MS/MS or structural isomers with identical MS/MS fragmentation. Low-molecular-weight and low-abundance metabolites typically have sparse MS/MS spectra and lack characteristic product ions for structural elucidation^48^. IMS collision cross-section (CCS) values are not yet included in the community standards for metabolite identifications, yet our CCS values improved the accuracy of metabolite annotation. Our CCS measurements^37^, based on molecular shape and size, aided in metabolite identifications and distinguished isomers. Unlike RT, CCS is highly reproducible across instruments and labs^49–51^. The power of MIPI is to leverage spatially constrained pathway-level data to support metabolite identifications.

In our MIPI analyses, each detected enzyme can produce many metabolites depending on the local pool of available substrates. Integration of MIPI data provides a comprehensive view of underlying biological pathways within the micrometer-scale environment, thereby providing enhanced confidence in individual metabolite and protein identifications when substrate, enzyme and product are colocalized. A current limitation of our MIPI workflow is relying on bulk measurements to provide MS/MS metabolite identifications. Providing spatially constrained microscale MS/MS measurements would further increase MIPI’s metabolite identification confidence. LESA–MS/MS provides spatial information but overall gives only one MS^1^ spectrum per pixel via direct infusion. To overcome these limitations, future efforts will couple nanoLC systems into the LESA–MS/MS setup by putting an upfront nanoLC separation (that is, HILIC) before MS detection. This will enable deeper metabolome profiling with confident molecular identification within each microhabitat.

## Methods

### Sample collection and laboratory-reared colonies

Fungal garden samples were collected from *A. cephalotes* colonies maintained in the laboratory. These colonies were reared on a mix of red maple (*Quercus rubra*) and red oak (*Acer rubrum*) leaves. Samples collected from the middle stratum of the garden were snap frozen once collected until further processing.

### Sample embedding and cryosectioning

The collected fungal garden sample was embedded in 7.5% hydroxypropyl methylcellulose and 2.5% polyvinylpyrrolidone and water and snap frozen in isopropanol chilled on dry ice to maintain fungal garden microintegrity. Embedding provided spatial compartmentalization, which allowed us to analyze unique features individually. The embedded sample was mounted on a cryomicrotome chuck by freezing a small droplet of water and then cut into 12-μm-thick sections using a CryoStar NX70 (Thermo Fisher) with a blade temperature of −18 °C and specimen temperature of −12 °C. Sections were thaw mounted onto indium tin oxide-coated glass slides (Bruker Daltonics), dried under vacuum and stored at −80 °C. After collecting three sections onto the indium tin oxide slide, four serial sections were placed on polyethylene naphthalate membrane slides (ZEISS) for subsequent laser-capture microdissection analysis. The same collection scheme was followed for the subsequent sample sectioning. Sections on polyethylene naphthalate slides were washed with a gradient of ethanol solutions (70, 96 and 100% ethanol, respectively) for 30 s during each change to dehydrate the sections and remove embedding material.

### Spatial metabolomics analyses using matrix-assisted laser desorption/ionization 15-T Fourier transform ion cyclotron resonance

An HTX TM-Sprayer (HTX Technologies) was used for *N*-(1-naphthyl)-ethylenediamine dihydrochloride (7 mg ml^–1^ in 70% methanol:water) MALDI matrix applications using the following spraying conditions: eight passes at 1,200 µl min^−1^ with spraying parameters of 75 °C, a spray spacing of 3 mm and a spray velocity of 1,300 mm min^−1^. MSI was performed in negative mode on a 15-T MALDI–FTICR mass spectrometer (Bruker Daltonics) equipped with a SmartBeam II laser source (355 nm, 2 kHz) and 50-µm pitch between pixels. The FTICR mass spectrometer was operated to collect ions with *m*/*z* values of 92 to 700 using a 209‐ms transient that translated to a mass resolution of *R* ~70,000 at 400 *m*/*z*. The instrument was internally calibrated using the MALDI matrix peak (*N*-(1-naphthyl)-ethylenediamine dihydrochloride) as a lock mass (*m*/*z* 256.776950) during the imaging run. Data were obtained using FlexImaging (v4.1, Bruker Daltonics), and image processing, segmentation, colocalization analysis and visualization were performed using SCiLS Lab (v2024a Core, Bruker Daltonics).

The MALDI–FTICR–MSI data were imported into the SCiLS Lab software (v2024a Core, SQLite format, Bruker Daltonics) and converted into the imzML format. The resulting imzML and ibd files were then uploaded to METASPACE (https://metaspace2020.eu) for molecular annotation and data visualization. This open cloud software platform performs annotation based not only on the accurate mass information but also on a comprehensive bioinformatics framework that considers the relative intensities and spatial colocalization of isotopic peaks and also quantifies spatial information with a measure of spatial chaos followed by estimation of the FDR^53^. Data were annotated using the KEGG database and are reported with an FDR of ≤20%.

### Spatial proteomics analyses using the microdroplet processing in one pot for trace samples approach

Using a PALM MicroBeam system (ZEISS), morphologically distinct features, previously identified by MALDI–MSI, were microdissected and collected in the corresponding microwells of the microPOTS chip that was preloaded with 3 µl of DMSO, which served as a capturing medium for excised tissue collection. Following our collection scheme, we dissected and separately collected three replicates of LM1, LM2 and PMM and six replicates of LM3 ROIs using adjacent fungal garden sections. Although the morphology gradually changed as the cryosectioning progressed, the feature in one of the LMs was still observable 200 µm apart from the initial section used for the metabolome profiling; therefore, six replicates were collected for LM3 ROIs.

The microPOTS chip and its cover were incubated at 75 °C for 1 h to dry the DMSO solvent. Next, 2 µl of extraction buffer containing 0.2% *n*-dodecyl-β-d-maltoside, 0.5× PBS, 50 mM triethylamonium bicarbonate (TEAB) and 1 mM dithiothreitol was dispensed into each well of the chip. The chip was incubated at 75 °C for 1 h. Thereafter, 0.5 µl of iodoacetamide solution (10 mM iodoacetamide in 100 mM TEAB) was added to the corresponding wells with the samples, followed by incubation at room temperature for 30 min. All samples were subsequently digested by adding 0.5 µl of an enzyme mixture (10 ng of Lys-C and 40 ng of trypsin in 100 mM TEAB) and incubated at 37 °C for 10 h. Following digestion, peptides were acidified by adding 5% formic acid (FA) to each sample to a final concentration of 1% FA. Each sample was collected and dispensed into a 30-µl aliquot of LC buffer A (water with 0.1% FA), centrifuged at 10,000*g* for 5 min at 25 °C and transferred (~25 µl) to an autosampler vial coated with 0.01% *n*-dodecyl-β-d-maltoside. To minimize droplet evaporation, during every manipulation of the sample, the microPOTS chip was placed on an ice pack. Also, during each incubation, the microPOTS chip was sealed with the chip cover, wrapped in aluminum foil and incubated in a humidified chamber.

Peptide mass analyses were performed using a Q Exactive Plus Orbitrap mass spectrometer (Thermo Scientific) coupled to a custom LC system that consisted of a PAL autosampler (CTC Analytics), two Cheminert six-port injection valves (Valco Instruments), a binary nanoUPLC pump (Dionex UltiMate 3000, Thermo Scientific) and an HPLC sample loading pump (1200 Series, Agilent). Data were obtained using Thermo Xcalibur (v4.0) and Exactive software (v2.8 SP1). The sample was fully injected into a 25-µl loop and loaded onto an SPE precolumn (150 µm inner diameter (i.d.), 5 cm length) using Buffer A at a flow rate of 3 µl min^−1^ for 30 min. Following SPE cleanup, the concentrated sample was backflushed on an LC column (50 µm i.d., 60-cm self-pack PicoFrit column, New Objective). Both SPE precolumns and columns were slurry packed with 5-µm and 3-µm Jupiter C18 packing material (300-Å pore size; Phenomenex), respectively. Chromatographic separation was performed at 200 nl min^−1^ using the following gradient: 1–8% (2.6 min–12.6 min), 8–25% (12.6 min–107 min), 25–75% (107 min–122.6 min) and 75–95% (122.6 min–125.9 min) Buffer B (0.1% FA in acetonitrile) followed by column washing and re-equilibration. Separated peptides were introduced to the ionization source in which high voltage (2,200 V) was applied to generate electrospray and ionize peptides. The ion transfer tube was heated to 300 °C, and the S-Lens RF level was set to 60. Data were acquired in positive mode. Full MS scans were acquired across a scan range of 300 to 1,800 *m*/*z* at a resolution of 70,000, combined with a maximum injection time of 20 ms and an automatic gain control target value of 3 × 10^6^. Twelve data-dependent MS/MS scans were recorded per MS scan at a resolving power of 17,500 combined with a maximum injection time of 50 ms and automatic gain control target value of 1 × 10^5^ with an isolation window of 2 *m*/*z*.

#### Customized protein database for peptide tandem mass spectrometry data annotation

A customized protein database was curated to annotate the MS/MS data. The schematic workflow of metaproteomics data analysis for taxonomic and functional assignments (Method 1) is depicted in Supplementary Fig. 1. The protein database consists of two types of protein sequences. The first type is proteins translated from the predicted genes in assemblies of the metagenomes of leaf-cutter ant gardens deposited at the Joint Genome Institute (JGI) data portal, as well as genomes of the resident microbes^6,11,44^. The second type is protein sequences predicted using the method of de novo metaproteomics, as described previously^33^. The customized protein database includes 147 FASTA files, and their detailed descriptions are shown in Supplementary Table 11. Protein sequences were dereplicated at 90% of identity using CD-HIT (v4.8.1; parameters applied: -c 0.9 -n 5 -G 1 -g 1 -b 20 -l 10 -s 0.0 -aL 0.0 -aS 0.0 -T 4 -M 32000), resulting in 24,809,256 non-redundant protein clusters. The protein sequence with the longest length within each cluster was selected as the representative sequence of the cluster and named ‘cluster protein’. The cluster proteins were annotated using the KEGG database via the ‘Functional Annotation’ module of the JGI metagenome workflow^54^ and Kofamscan (v1.2.0; thresholds applied: e value < 10^−5^ and bit score > 50). The taxonomic annotation of the cluster protein was assigned using the ‘Taxonomic annotation’ module of the JGI metagenome workflow^54^.

#### Peptide tandem mass spectrometry data analysis

MS/MS data were searched against our customized protein database with a decoy database of reversed sequences using MS-GF+^55^ (v2021.09.06) and MASIC (released 3.2.7901). Only PSMs with a maximum of two missed trypsin cleavages and zero irregular cleavages (NTT = 2) were kept for further FDR calculations. The spectrum-level peptide confidence score of the PSM (that is, MSGFDB_SpecProb) and mass difference (in ppm) between the observed parent ion and the computed mass of the identified peptide (that is, DelM_PPM in MS-GF+) values were optimized to achieve the highest number of peptide identification within each dataset while keeping the target–decoy-based FDR of both peptide identifications and spectrum identifications below 5%. The absolute adjusted mass errors (that is, DelM_PPM) were less than 4.5, and the MSGFDB_SpecProb values of >91% of the final PSMs were less than 1 × 10^–10^. These data were further filtered to include only qualified peptides with at least two PSMs identified and exclude proteins with less than two qualified peptides identified.

Methods complementary to JGI workflows (functional and taxonomic assignment, Method 1, Supplementary Fig. 1) were applied to ensure confident functional and taxonomic assignments to the quality-filtered peptides that matched the cluster proteins. The cluster protein with multiple peptides matched was searched against the National Center for Biotechnology Information (NCBI) clustered non-redundant (NR) database (accessed on 17 February 2023) using BLAST (v2.13.0+). The top ten hits to the cluster protein were recorded, and the hits with bit scores of >50 and e values of <10^−5^ were examined. The cluster protein was assigned with the functional annotation that the top hits with the same highest bit score agreed on and otherwise remained unclassified by the NCBI clustered NR database (functional assignment, Method 2, Supplementary Fig. 1). In addition to the JGI metagenome workflow, the taxonomic assignment of the cluster proteins was made using both protein sequence-based (taxonomic assignment, Method 3, Supplementary Fig. 1) and contig-based methods (taxonomic assignment, Method 2, Supplementary Fig. 1). The protein sequence-based method followed a similar rule as described in the functional annotation section. The cluster protein was assigned with the taxonomy with the top hits having the same highest bit score agreed upon or the lowest common ancestor of these top hits, which otherwise remained unclassified by the NCBI clustered NR database. Contigs where the cluster proteins were predicted via the ‘Feature prediction’ module of the JGI metagenome workflow were retrieved from the respective metagenomes. The taxonomy of the contig was annotated using the Contig Annotation Tool (CAT, v5.2.3) that applied a voting-based classification rule on the multiple open reading frames of the contig. The functional and taxonomic annotation results of the peptide-matched cluster proteins using the complementary methods were cross-checked with the respective records obtained through the JGI metagenome workflow (Supplementary Table 12).

### Metaproteome and metabolome data integration

We integrated metaproteomic and metabolomic evidence to confidently reconstruct the metabolic pathways of the studied microbiomes. The KO identifiers of the detected cluster proteins were queried against the KEGG database to fetch all the associated KEGG reactions using ‘keggLink’ implemented in KEGGREST (v1.38.0). The related compounds of these KEGG reactions were recovered after cross-referencing the KEGG reaction and compound databases using KEGGREST (v1.38.0). The resulting compound identifiers and descriptions were searched for the metabolites identified in the paired metabolome datasets.

The KEGG database documents the known relationship of compound–enzyme–reaction–pathway–metabolic category/module using model organisms, including fungi (https://www.genome.jp/kegg-bin/show_organism?menu_type=pathway_maps&category=Fungi). This is how we link the compounds detected by the metabolome and the enzymes detected by the paired metaproteome and assign the molecular annotations to pathway and metabolic categories.

### Statistics and reproducibility

Two MALDI image replicates (sections 1 and 9) reproducibly detected the same spatial arrangement of microhabitats LM and PMM, although collected approximately 100 µm apart. Informed by the metabolome-specific features, three distinct LM ROIs (LM1, LM2 and LM3) and one PMM ROI were further investigated at the proteome level. Sections in close proximity to each of the two MALDI-imaged fungal garden sections retained the same spatial arrangement of these microhabitat features. These sections, therefore, were considered replicates of each other. LM1, LM2 and PMM had three replicates (R1–R3), that is, LM1 R1, LM1 R2, LM1 R3, LM2 R1, LM2 R2, LM2 R3, PPM R1, PPM R2 and PPM R3. LM3 was a larger microhabitat along the depth gradient with six sections (R1–R6), that is, LM3 R1, LM3 R2, LM3 R3, LM3 R4, LM3 R5 and LM3 R6.

Correlation metrics between metaproteome and metabolome data were calculated between colocalized peptides and metabolites for pathways of interest. Individual correlation values and matching adjusted *P* values to each peptide to substrate and product metabolites can be found in Supplementary Table 14. Sufficient data (that is, at least two observed intensities in at least three replicate sections of each microhabitat) were required to compute a metric. For each peptide, log_2_ (fold change) values of mean normalized intensities for all pairwise microhabitats were computed; when insufficient data existed in one group to compute a mean, a directional flag of significance, based on a G-test^56^, was used for the pairwise comparison. Similarly, for each *m*/*z*, the log_2_ (fold change) value of mean pixel intensities within each region of interest was computed. For each pairwise comparison metric, data were first centered and scaled to a unit variance, and the Pearson correlation between each peptide and *m*/*z* image was then computed. A test of significance for each computed correlation was then conducted, and a Benjamini–Hochberg^57^ multiple-testing *P* value adjustment was conducted. R (v3.2.1) was used for statistical analyses.

### MS^2^-based metabolite identifications

Because MALDI–MSI uses an MS^1^-based approach for metabolite annotation, various orthogonal techniques were used on the biological replicates of the fungal garden middle stratum to obtain comprehensive and accurate molecular characterization.

The middle stratum of the fungal garden sample was lyophilized for the untargeted bulk metabolomics analysis. To break up the biological material, the sample was bead beaten with a 3-mm tungsten carbide bead at 1,400 c.p.m. for 2 min using a 2010 Geno/Grinder Automated Tissue Homogenizer and Cell Lyser (SPEX) using cooled cryoblocks to inhibit sample heating. Metabolites were extracted from approximately 20 mg by resuspending the sample in 470 μl of an ice-cold 4:3 (vol/vol) methanol:water mixture, adding 530 μl of ice-cold chloroform, vortex mixing for 1 min, incubating on ice for 5 min and vortex mixing for 1 min. The sample was centrifuged at 10,000*g* at 4 °C for 10 min. The top phase was transferred to the vials and analyzed using (1) GC–MS^8^, (2) LC–MS/MS using RP^58^ and HILIC^59^ separation methods and data-dependent acquisition and (3) LC–IMS–MS/MS using HILIC separation methods^37,59^ and All Ions DIA with 20-V collision energy, as previously described^37^. To complement these LC–MS/MS methods, (4) LESA provided MS/MS directly from fungal garden sections. Leveraging these four complementary metabolomics techniques provided MS^2^ identifications of diverse metabolites.

#### Gas chromatography–mass spectrometry

Metabolite extracts were dried completely under vacuum. Dried extracts were chemically derivatized as previously reported^60^. The extracted metabolites were derivatized by methoxyamination and trimethylsilyation, and the samples were analyzed by GC–MS. Samples were run in an Agilent GC 7890A using an HP-5MS column (30 m × 0.25 mm × 0.25 μm; Agilent Technologies) coupled with a single quadrupole MSD 5975C (Agilent Technologies). One microliter of sample was injected into a splitless port at a constant temperature of 250 °C. The GC temperature gradient started at 60 °C, with a hold of temperature for 1 min after injection, followed by an increase to 325 °C at a rate of 10 °C min^–1^ and a 10-min hold at this temperature. A fatty acid methyl ester standard mix (C8–C28; Sigma-Aldrich) was analyzed in parallel as a standard for RT calibration. GC–MS raw data files were processed using Metabolite Detector software^61^. Retention indices of detected metabolites were calculated based on the analysis of a FAMEs mixture, followed by their chromatographic alignment across all analyses after deconvolution. Metabolites were initially identified by matching experimental spectra to a Pacific Northwest National Laboratory (PNNL) augmented version of the Agilent GC–MS metabolomics library containing spectra and validated retention indices for over 1,200 metabolites. The unknown peaks were then additionally matched with the NIST20/Wiley11 GC–MS library.

#### Reversed-phase and hydrophilic interaction liquid chromatography-tandem mass spectrometry

In short, for LC–MS/MS, RP separation was conducted using a Thermo Hypersil GOLD column (2.1 × 150 mm, 3-μm particle size) with a column temperature of 40 °C and a flow rate of 400 μl min^–1^. Mobile phases A (water with 0.1% FA) and B (acetonitrile with 0.1% FA) were initially 90:10, respectively. The gradient method continued using the following parameters: 0–2 min hold at 90% A; 2–11 min 10% A; 11–12 min 10% A; 12–12.5 min hold at 10% A with an increased flow rate to 500 μl min^–1^; 12.5–13.5 min at 90% A, flow rate at 500 μl min^–1^; 13.5–14 min at 90% A, flow rate at 500 μl min^–1^; 14–14.5 min at 90% A, decrease flow rate to 400 μl min^–1^ and 14.5–16 min hold at 90% A. HILIC separation was conducted using an ACQUITY UPLC BEH HILIC column (2.1 × 100 mm, 1.7-μm particle size) with a column temperature of 50 °C and a flow rate of 300 μl min^–1^. Mobile phases A (5% acetonitrile and 95% 10 mM ammonium acetate in water with 0.05% ammonium hydroxide) and B (100% acetonitrile with 0.05% ammonium hydroxide) were initially 5% and 95%, respectively, at a flow rate of 300 μl min^–1^. The gradient method continued with the following parameters: 0–6.0 min 63% A; 6.0–7.0 min 63% A; 7.0–7.1 min 5% A; 7.1–7.2 min 5% A with an increased flow rate of 500 μl min^–1^; 7.2–9.5 min hold at 5% A; 9.5–9.7 min 5% A with a decreased flow rate of 300 μl min^–1^ and 9.7–12.0 min hold at 5% A. All data were collected on a Q Exactive Plus in both positive and negative ionization modes. Full MS scan data were acquired at a resolving power of 140,000 full-width at half-maximum at *m*/*z* 200 with a scanning range of *m*/*z* 80–800. The data-dependent acquisition parameters used to obtain product ion spectra were collected at a resolving power of 17,500 full-width at half-maximum at *m*/*z* 200 and normalized collision energy of 20, 30 and 40 V. For RP and HILIC positive and negative mode data, spectra were aligned using an adaptive curve with a maximum of 0.3 RT shift and a 3-ppm mass tolerance. Peaks were selected based on a minimum intensity of 1 × 10^6^ and a chromatographic signal-to-noise ratio of 3. Detected features were grouped based on a mass tolerance of 3 ppm and an RT tolerance of 0.25. Features that were not present in at least four samples were filtered out. Metabolites were matched based on RT where applicable, isotopic ratio, monoisotopic mass (within 5 ppm) and MS/MS match to internal and external databases, including MzCloud and Mass Bank of North America. Compound Discoverer (v3.3) was used for metabolite identification. All identifications and integrated peaks were manually validated and exported for statistical analysis.

#### Liquid chromatography–ion mobility spectrometry–tandem mass spectrometry

For LC–IMS–MS/MS, chromatographic separation was performed with an Agilent UHPLC 1290 Infinity II system coupled to an Agilent 6560 Drift Tube Ion Mobility Spectrometer. The sample injection volume was 10 µl, and the autosampler temperature was maintained at 4 °C. The Agilent UHPLC was equipped with a Water XBridge BEH Amide XP column (2.5 µm; 2.1 mm i.d. × 50 mm). A Waters XBridge BEH Amide XP VanGuard cartridge (2.5 µm; 2.1 mm i.d. × 5 mm) was also installed to remove potential particulate contamination from the mobile phases. HILIC chromatographic separation was conducted using the aforementioned HILIC separation method and the following method. Mobile phase A consisted of 10 mM ammonium acetate and 10 µM InfinityLab deactivator additive (pH 9.2) in 90% water and 10 % acetonitrile, and mobile phase B consisted of 10 mM ammonium acetate (pH 9.2) in 90% acetonitrile. The column was maintained at 50 °C throughout the run. The gradient length was 8.70 min (in detail, 0.0:0.350:90, 1.0:0.350:90, 1.1:1.0:85, 4.0:0.750:80, 5.0:0.750:40, 6.5:0.750:40, 6.8:0.750:20, 7.0:0.750:20 and 7.5:0.750:90 in terms of min:flow rate (in ml min^–1^):% B) with an equilibration time of 3.0 min. For LC–IMS–MS data processing, the PNNL-Preprocessor^62^ (v2020.07.24) was used to apply moving average smoothing (points: 3 in LC and 3 in IM) and filtering (minimum intensity threshold of 20 counts). LC–IMS–MS feature detection and CCS calibration were performed using the IM-MS Browser (v.10.0, Agilent Technologies) with the single-field method and the Agilent Tune-Mix solution as calibrants. A list of target ions was generated using exact masses matched to detected MS^1^ features and fragment ions from predicted MS/MS spectra by CFM-ID^63^ (v4.0). Skyline^64^ (v.64.21.1.0.146) was used to perform targeted data extraction (acquisition method DIA, isolation scheme All Ions, mass analyzer TOF, mass resolving power 10,000, ion mobility resolving power 40 and small-molecule fragment types ‘p,f’)^37^.

#### Liquid extraction surface analysis–tandem mass spectrometry

Biological replicates from the fungal garden middle stratum were embedded in hydroxypropyl methylcellulose/polyvinylpyrrolidone matrix and cryosectioned following the aforementioned protocol (Sample embedding and cryosectioning). Sections of 20 µm in thickness were obtained and scanned for morphology assessments. Regions that were morphologically similar to the regions imaged using the MIPI approach (LM1–LM3) were analyzed using LESA–MS/MS techniques (Supplementary Fig. 6). Metabolites were extracted from the sample surface with 0.5 µl of 70% methanol with 0.1% FA and were directly infused into the analyzer (Q Exactive Plus Orbitrap), as previously described^26^. The in silico fragmenter MetFrag^65^ (https://ipb-halle.github.io/MetFrag/) was used to score molecular identification of isomeric species from the KEGG database based on experimental MS/MS spectra.

### Reporting summary

Further information on research design is available in the Nature Portfolio Reporting Summary linked to this article.

## Online content

Any methods, additional references, Nature Portfolio reporting summaries, source data, extended data, supplementary information, acknowledgements, peer review information; details of author contributions and competing interests; and statements of data and code availability are available at 10.1038/s41589-023-01536-7.

### Supplementary information

Supplementary Information Supplementary Figs. 1–6 and Tables 6–9.

Reporting Summary

Supplementary Tables Supplementary Tables 1–5 and 10–14.

## Data Availability

MALDI–MSI data generated for sections 1 and 9 can be found at https://metaspace2020.eu/annotations?db_id=374&prj=e6a0f848-d586-11ed-afbb-1b53f1cbcdef&ds=2021-09-20_21h49m04s&fdr=0.2&sort=fdr_msm&sections=5&row=2 (section 1) and https://metaspace2020.eu/annotations?db_id=374&prj=e6a0f848-d586-11ed-afbb-1b53f1cbcdef&ds=2021-09-21_23h56m53s&fdr=0.2&row=2 (section 9). Raw proteomics data can be found via the Mass Spectrometry Interactive Virtual Environment (MassIVE; https://massive.ucsd.edu) under accession number MSV000091701). The protein database was deposited at Zenodo at 10.5281/zenodo.7826514.
