# Supplementary material for: Mapping microhabitats of lignocellulose decomposition by a microbial consortium
Source: Nat Chem Biol. 2024 Feb 1;20(8):1033–43. doi: 10.1038/s41589-023-01536-7 (PMC11288888; doi:10.1038/s41589-023-01536-7)
Supplement: Supplementary file 1 — Supplementary Figs. 1–6 and Tables 6–9. [file 41589_2023_1536_MOESM1_ESM.pdf]

nature chemical biology

Article

https://doi.org/10.1038/s41589-023-01536-7

Mapping microhabitats of lignocellulose decomposition by a microbial consortium

In the format provided by the authors and unedited

Supplementary Information (SI)

Supplementary Figure 1. Schematic workflow of metaproteomic methods used for taxonomic and functional assignments … *page 2-3*

Supplementary Figure 2. Complementary MS2-based metabolite identifications of the metabolites from pathways of interest … *page 4-17*

Supplementary Figure 3. Multi-omics data integration using metabolite ion images obtained for replicate Section 9 … *page 18-19*

Supplementary Figure 4. A workflow with all the steps of the annotation and identification of fuconate in the MALDI-FTICR data … *page 20*

Supplementary Figure 5. Metabolites and isomers of the homogentisic pathway of homogentisate ring cleavage by fungus detected by LC-IMS-MS/MS … *page 21*

Supplementary Figure 6. Optical image of the fungal garden section with ROIs that were sampled using orthogonal LESA-MS/MS technique … *page 22*

Supplementary Table 6. Metabolites confirmed by annotated LC-IMS-MS features from the fungal garden sample … *page 23*

Supplementary Table 7 (in combined SI file). Metabolites confirmed by annotated LC-MS/MS features from the fungal garden sample … *page 24*

Supplementary Table 8 (in combined SI file). Metabolites confirmed by annotated LESA-MS/MS features from the fungal garden sample … *page 25*

Supplementary Table 9 (in combined SI file). Metabolites confirmed by annotated GC-MS features from the fungal garden sample … *page 25*

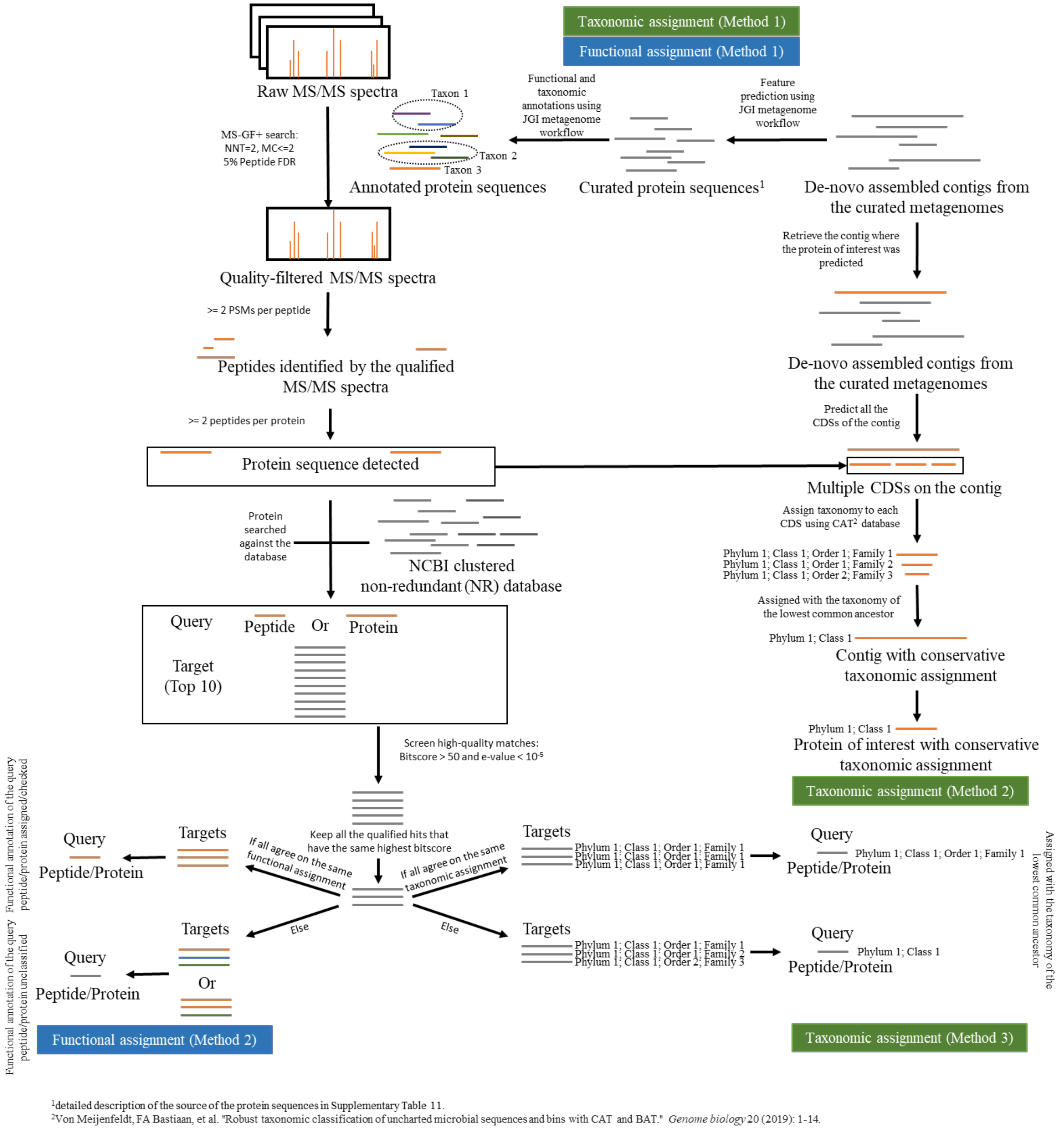

Supplementary Figure 1. Schematic workflow of metaproteomics data analysis for taxonomic and functional assignments. Taxonomic annotation: in addition to the taxonomic annotation module of the JGI metagenome workflow (Taxonomic assignment, Method 1), we applied two complementary methods, a contig-based method (Taxonomic assignment, Method 2) and a protein-based method (Taxonomic assignment, Method 3). In the protein-based method, the protein in our curated database that was mapped with no less than two quality-filtered peptides was subjected to taxonomic annotations. The protein was assigned with the taxonomy of the lowest common ancestor of the top hits in the NCBI clustered NR database having the same highest bitscore (min. bitscore of 50 and max. e-value of 10-5). The protein remained unclassified when no lowest common ancestor was identified at the Domain level. In the contig-based method, contigs, where the cluster proteins were predicted via the “Feature prediction” module of the JGI metagenome workflow, were retrieved from the respective metagenomes. The taxonomy of the contig was annotated using Contig Annotation Tool (CAT, v5.2.3) that applied a voting-based classification rule on the multiple open reading frames of the contig. Taxonomic assignment Methods 2 and 3 complement the JGI method (Method 1) which is based on a single top hit to allow

more conservative taxonomic assignment. In addition, we keep the taxonomic assignment at a higher rank (e.g., fungi, bacteria, insect) to avoid potential overlaps between different taxonomic groups. Functional assignment: In addition to JGI metagenome workflow (Functional assignment, Method 1), the protein (with >= 2 quality-filtered peptides) was assigned with the functional annotation that the top hits with the same highest bitscore agreed on, otherwise remained unclassified by NCBI clustered NR database (Functional assignment, Method 2).

Supplementary Figure 2. Complementary MS2-based metabolite identifications of the metabolites from pathways of interest.

Analyses

- Agilent 6560 Drift Tube Ion Mobility Q-TOF Mass Spectrometer - Liquid Chromatography-Ion Mobility Spectrometry-MS/MS (**LC-IMS-MS/MS**)
- Thermo Q Exactive Plus Orbitrap **-** Liquid chromatography-tandem MS (**LC-MS/MS**)
- Thermo Q Exactive Plus Orbitrap - Liquid extraction surface analysis-tandem MS (**LESA-MS/MS**)
- Agilent single quadrupole MSD 5975C - Gas Chromatography-Mass Spectrometry (**GC-MS**)

Identification confident metrics: Level 1 (high) to Level 5 (low)

Schymanski, E. L. *et al.* Identifying Small Molecules via High Resolution Mass Spectrometry: Communicating Confidence. *Environmental Science & Technology* **48**, 2097-2098 (2014). https://pubs.acs.org/doi/10.1021/es5002105

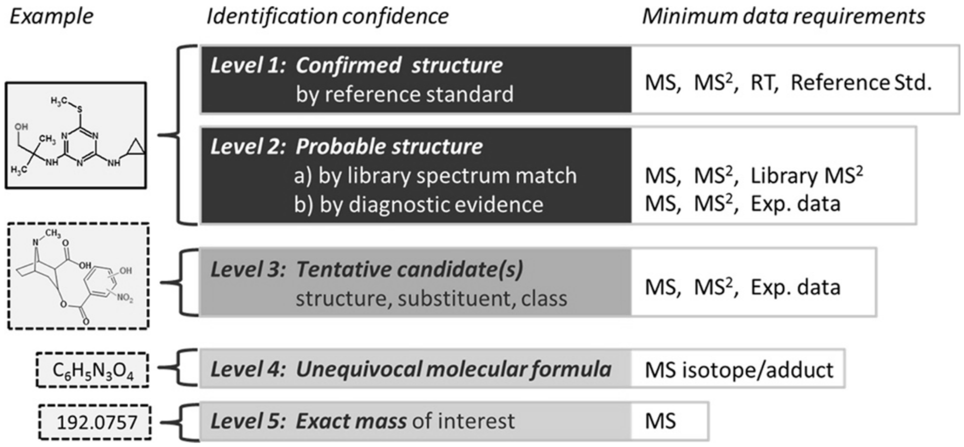

Figure 1. Identifying small molecules with confidence. Reprinted (adapted) with permission from Environ. Sci. Technol. 2014, 48, 4, 2097–2098. Copyright 2014 American Chemical Society.

- **Collision Cross Sections (CCS) from Differential Ion Mobility Spectrometry and pathway level support (i.e., complementary enzyme and product identifications within the same microscale region), although listed below, were not included in our reported identification confidence metrics for each metabolite.**

Metabolites

a) 4-Hydroxyphenylglyoxylate … *Page 5*
b) 4-Hydroxybenzaldehyde … *Page 6*
c) 4-Hydroxybenzoate … *Pages 7 and 8*
d) $[C_6H_4O_4 -H]^-$ This molecular formula matches to candidate molecules 4-Carboxymethylenebut-2-en-4-olide and 5-Formyl-2-furoate … *Page 9*
e) Benzene-1,2,4-triol … *Page 10*
f) 2-Maleylacetate … *Page 11*
g) Homogentisate … *Page 12*
h) 4-Maleylacetoacetate … *Page 13*
i) Fuconate … *Page 14*
j) 2-Dehydro-3-deoxy-fuconate … *Page 15*
k) Glucose … *Page 16*
l) Glucose 6-phosphate … *Page 17*

a) 4-Hydroxyphenylglyoxylate

i) <u>LC-IMS-MS/MS</u> - Identified with confidence **level 3** by accurate mass, retention time (RT), CCS, and MS/MS match with predicted spectra using CFM-ID (see Method Section for reference).

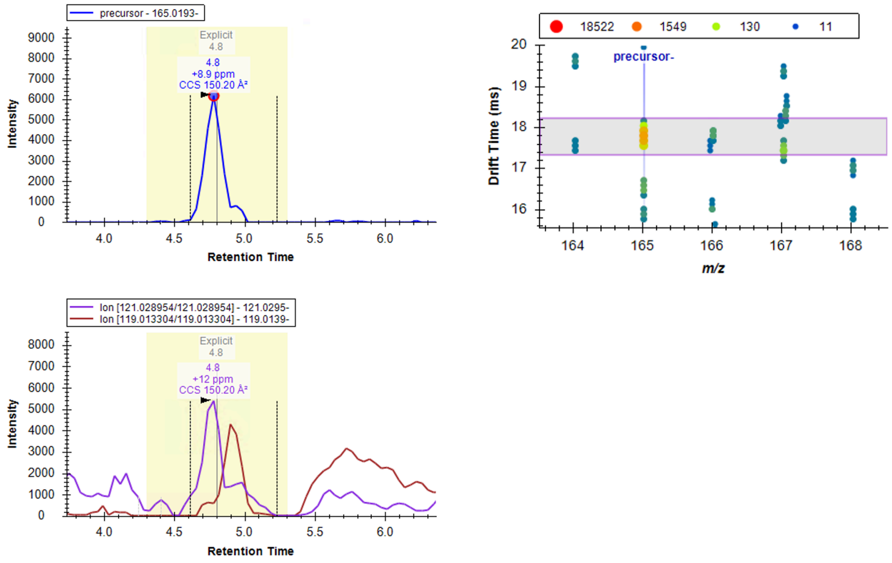

ii) <u>LC-MS/MS</u> - Identified with confidence **level 3** by accurate mass and MS/MS that makes sense for the ID.

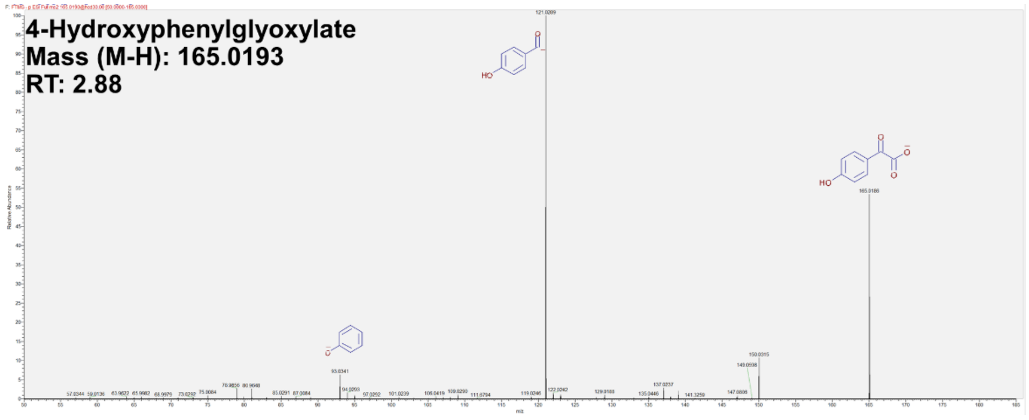

iii) <u>LESA-MS/MS</u> - Identified with confidence **level 3** by accurate mass and MS/MS that makes sense for the ID.

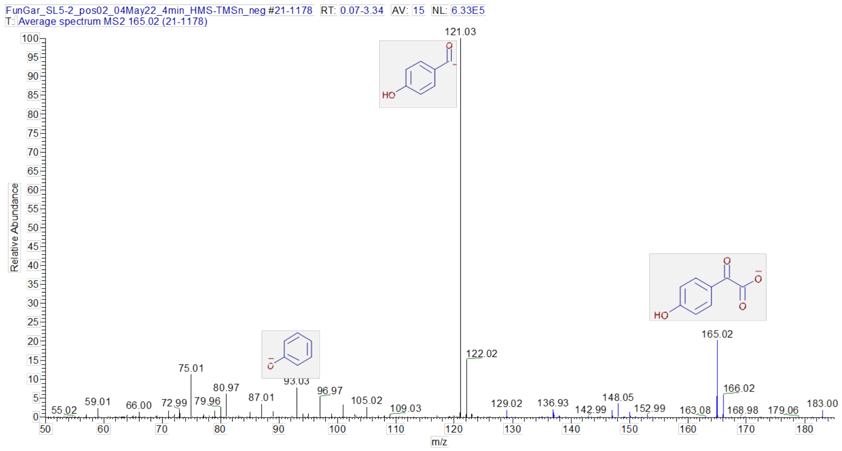

b) 4-Hydroxybenzaldehyde

i) <u>LC-IMS-MS/MS</u> – Identified with confidence **level 1** by accurate mass, RT, CCS, and MS/MS match with the standard.

STANDARD **SAMPLE**

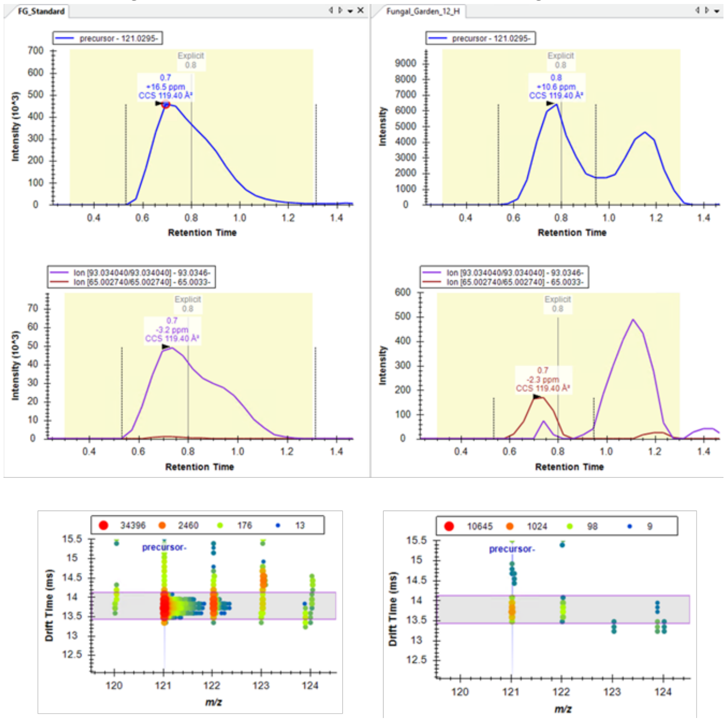

ii) <u>LC-MS/MS</u> - Identified with confidence **level 1** by accurate mass, RT, and MS/MS match with the standard.

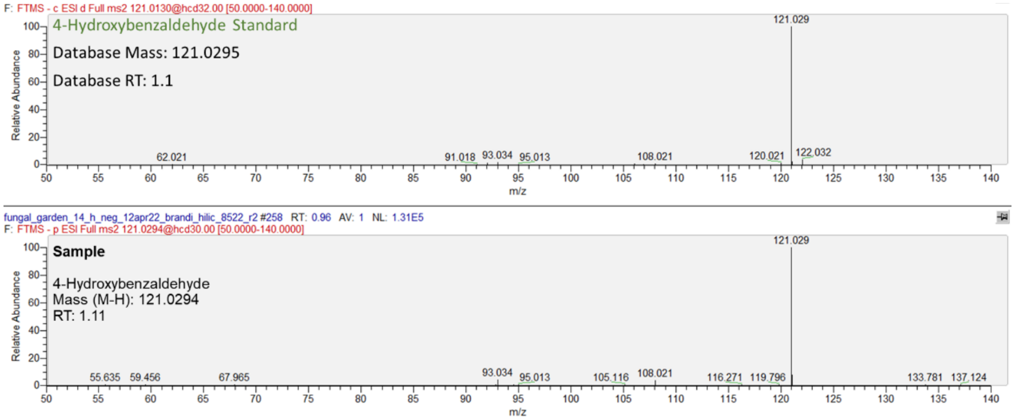

iii) <u>LESA-MS/MS</u> - Identified with confidence **level 3** by accurate mass and MS/MS that makes sense for the ID.

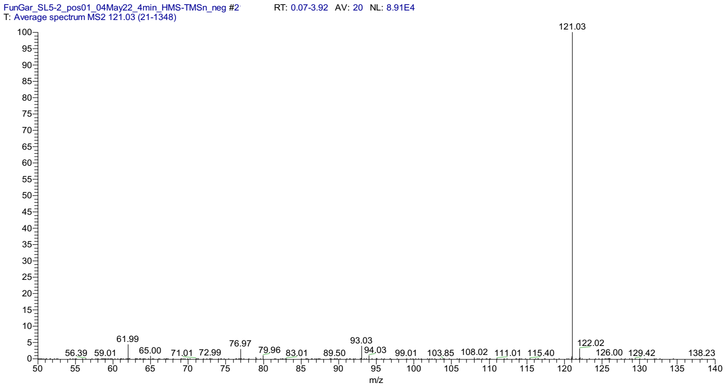

c) 4-Hydroxybenzoate

i) LC-IMS-MS/MS - Identified with confidence **level 1** by accurate mass, RT, CCS, and MS/MS match with the standard.

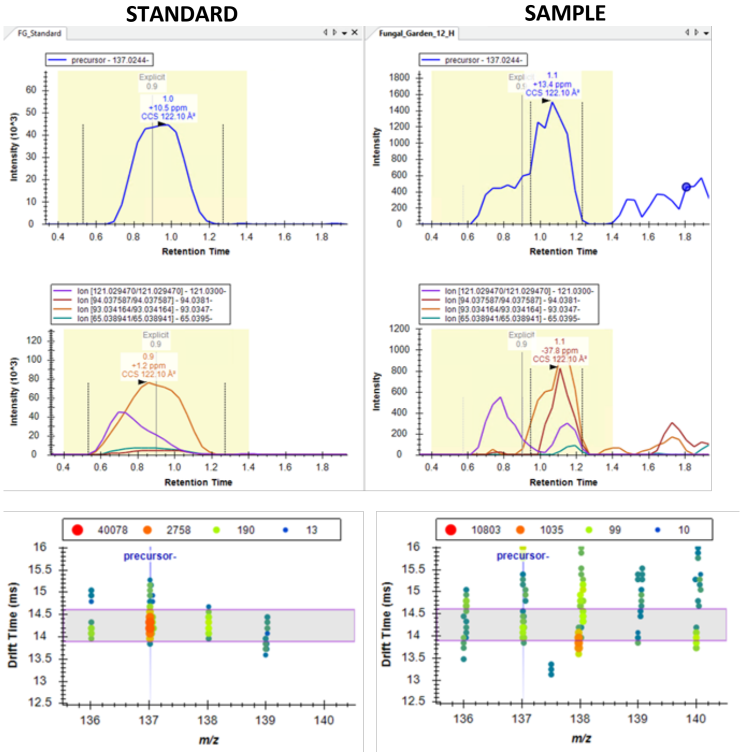

ii) LC-MS/MS - Identified with confidence **level 2** by identification by accurate mass, RT, and MS/MS match with the standard.

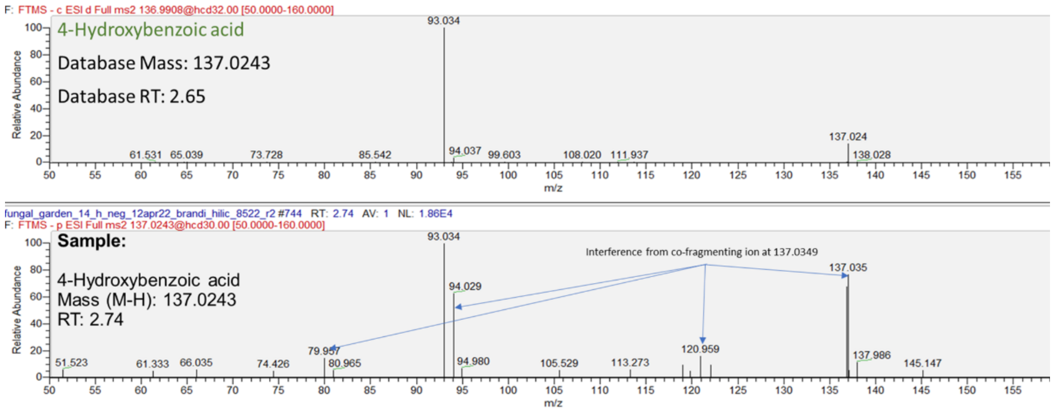

iii) **LESA-MS/MS** - Identified with confidence **level 3** by accurate mass and MS/MS that makes sense for the ID.

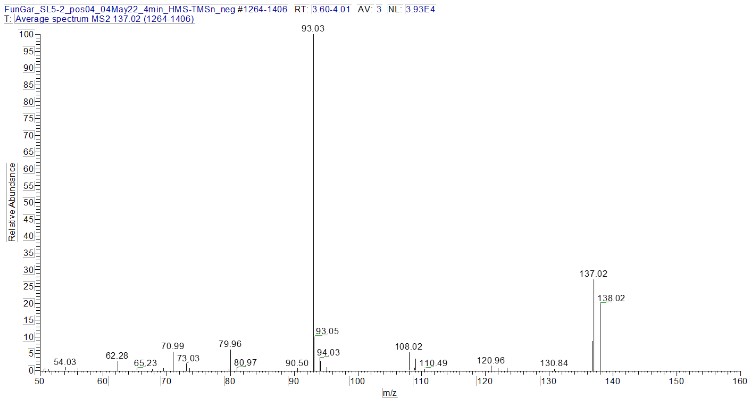

iv) **GC-MS** – Identified with confidence **level 2**. Retention index from sample 1314.4, retention index in standard: 1315.4. Fragments used to validate identification: 193, 223 and 267. The metabolite is co-eluting with other compounds that generate the adjacent ions observed in the MS.

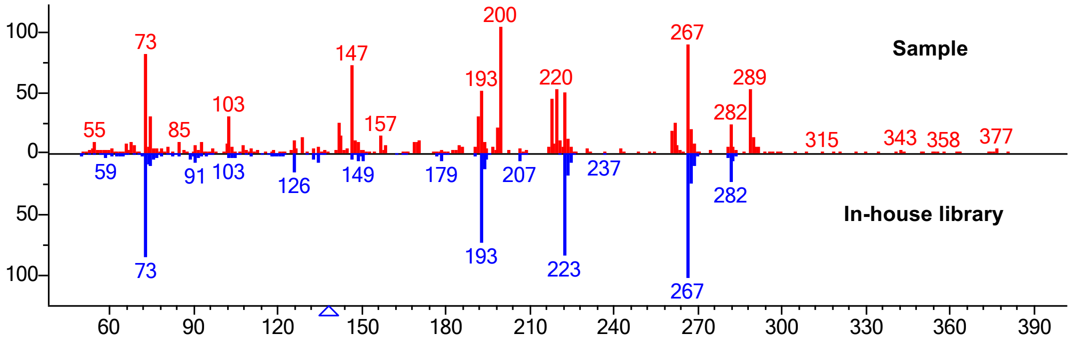

d) [$C_6H_4O_4$ -H]$^-$ This molecular formula matches to candidate molecules 4-Carboxymethylenebut-2-en-4-olide and 5-Formyl-2-furoate in the Kyoto Encyclopedia of Genes and Genomes (KEGG) database.

Annotation of 4-Carboxymethylenebut-2-en-4-olide is supported by the identification of the enzyme (Carboxymethylenebutenolidase) and product (2-Maleylacetate) within the same microscale regions (LM1, LM2, LM3).

i) LC-MS/MS – Identified with confidence **level 4** by accurate mass and isotope information.

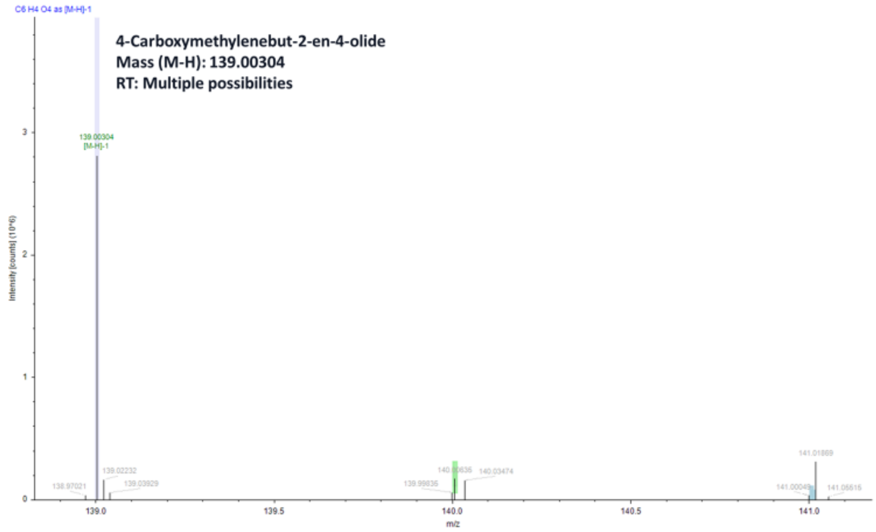

ii) LC-IMS-MS/MS - Identified with confidence **level 5** by accurate mass.

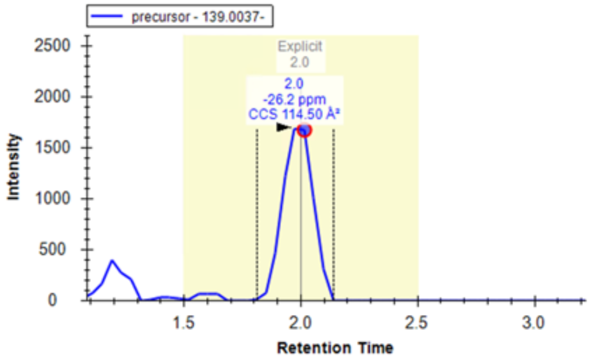

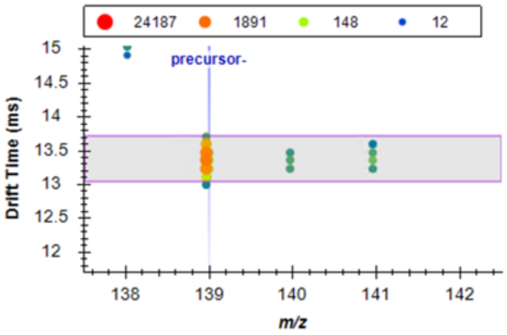

e) Benzene-1,2,4-triol

i) LC-IMS-MS/MS - Identified with confidence **level 3** by accurate mass, RT, CCS, and MS/MS match with predicted spectra using CFM-ID.

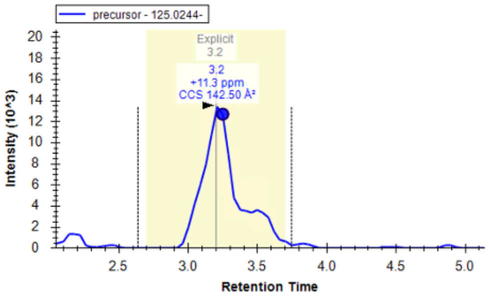

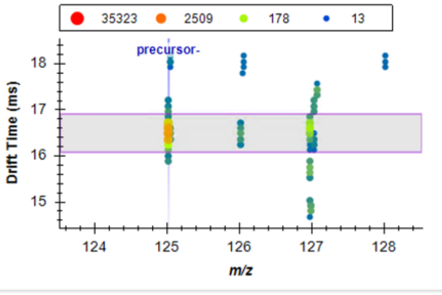

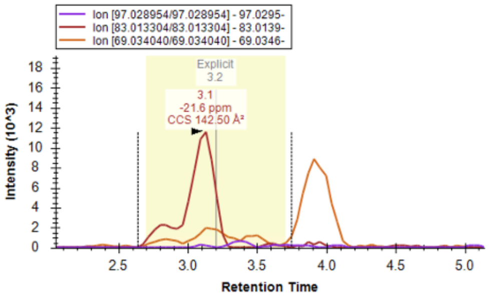

ii) LC-MS/MS - Identified with confidence **level 3** by accurate mass and MS/MS with external databases.

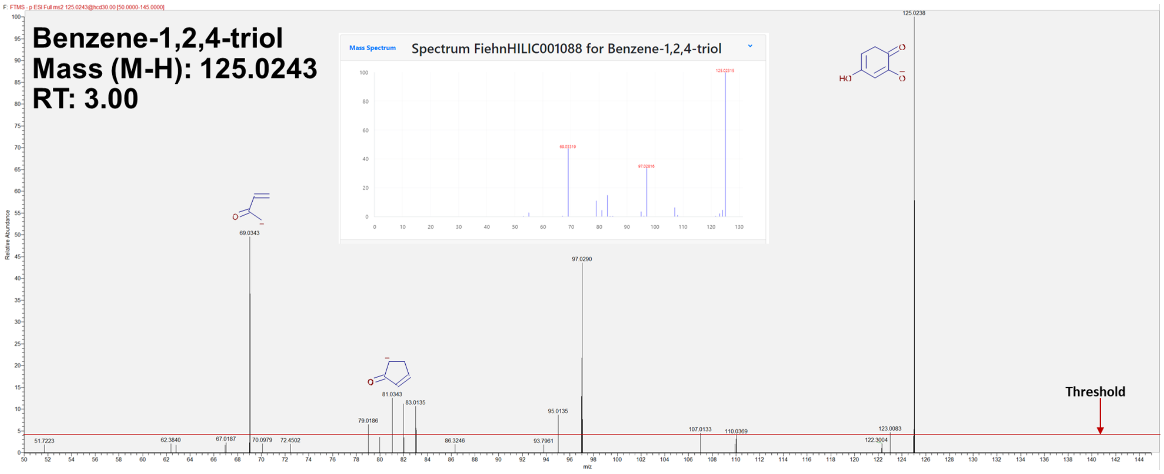

f) 2-Maleylacetate

i) LC-IMS-MS/MS - Identified with confidence **level 3** by accurate mass, RT, CCS, and MS/MS match with predicted spectra using CFM-ID.

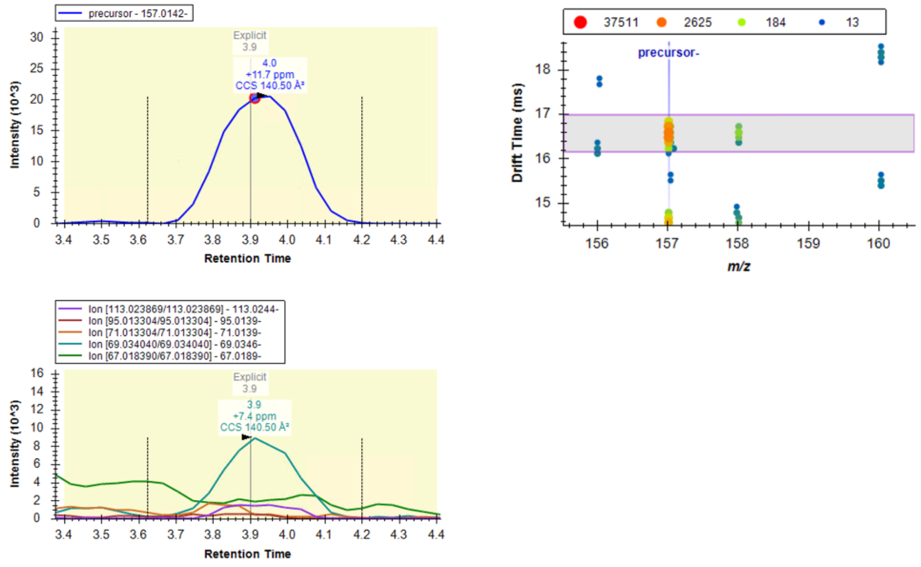

ii) LC-MS/MS - Identified with confidence **level 3** by accurate mass and MS/MS that makes sense for the ID.

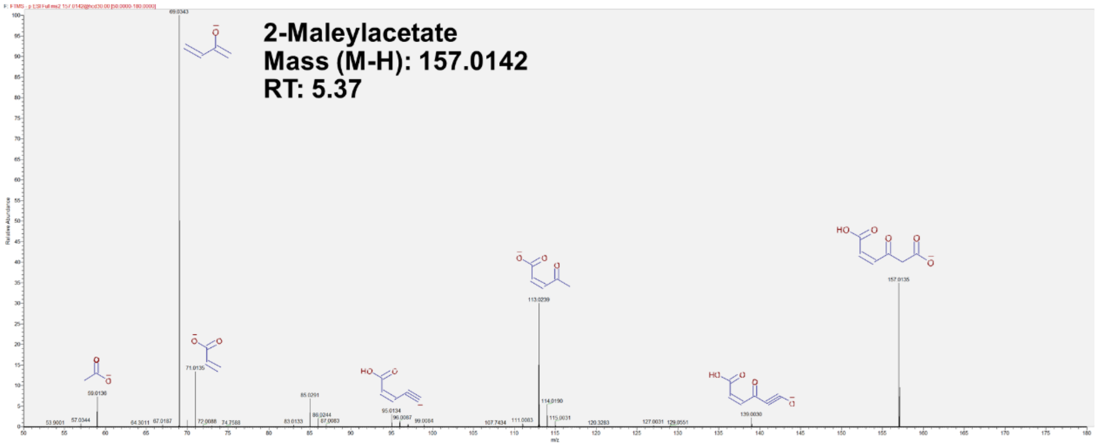

g) Homogentisate

i) **LC-IMS-MS/MS** - Identified with confidence **level 1** by accurate mass, RT, CCS, and MS/MS match with the standard.

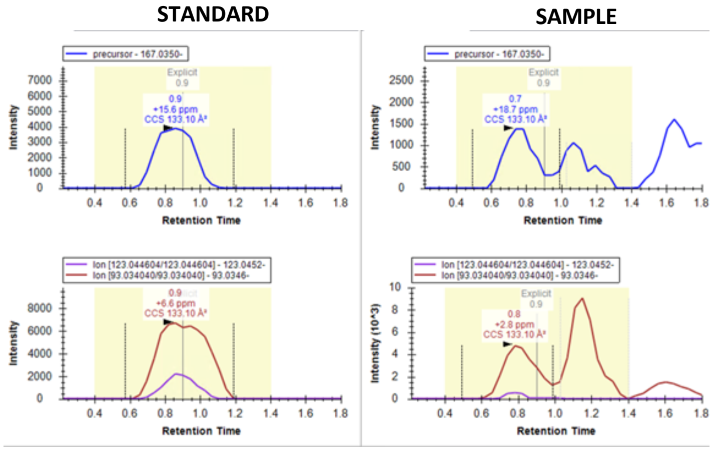

h) 4-Maleylacetoacetate

i) LC-MS/MS – Identified with confidence **level 4** by accurate mass and isotope information.

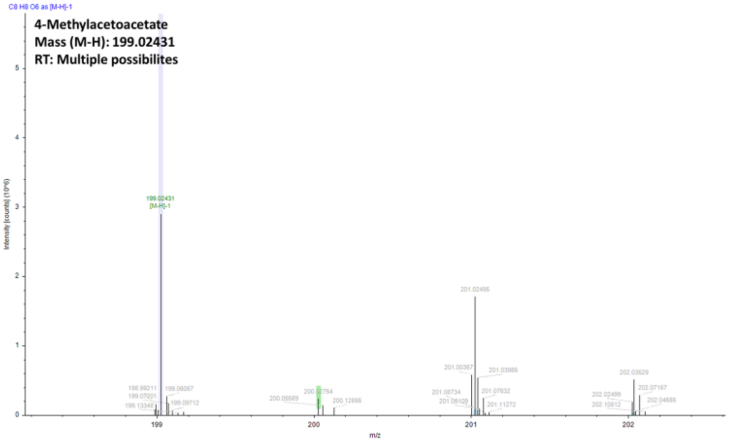

ii) LC-IMS-MS/MS - Identified with confidence **level 3** by accurate mass, RT, CCS, and MS/MS match with predicted spectra using CFM-ID.

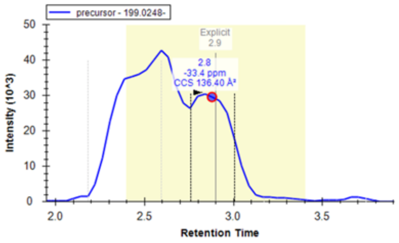

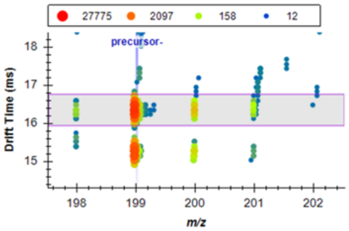

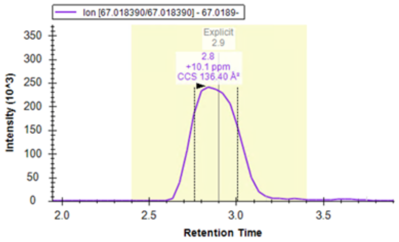

i) Fuconate

i) LC-IMS-MS/MS - Identified with confidence **level 5** by accurate mass.

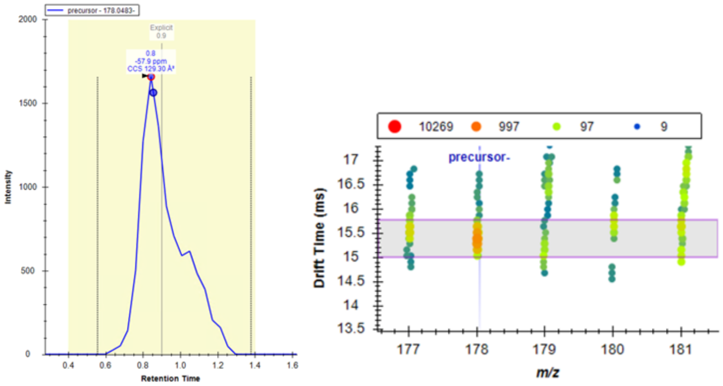

ii) LC-MS/MS - Identified with confidence **level 3** by accurate mass and MS/MS that makes sense for the ID.

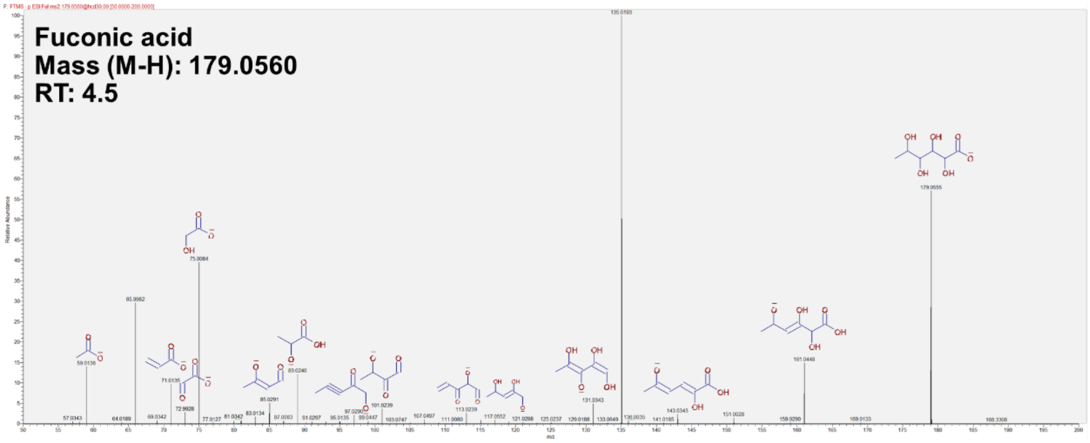

j) 2-Dehydro-3-deoxy-fuconate

i) LC-IMS-MS/MS - Identified with confidence **level 5** by accurate mass.

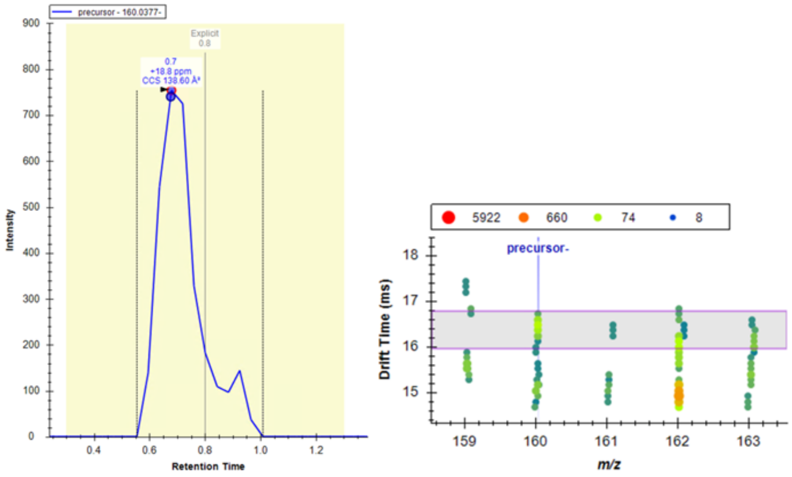

ii) LC-MS/MS - Identified with confidence **level 3** by accurate mass and MS/MS that makes sense for the ID.

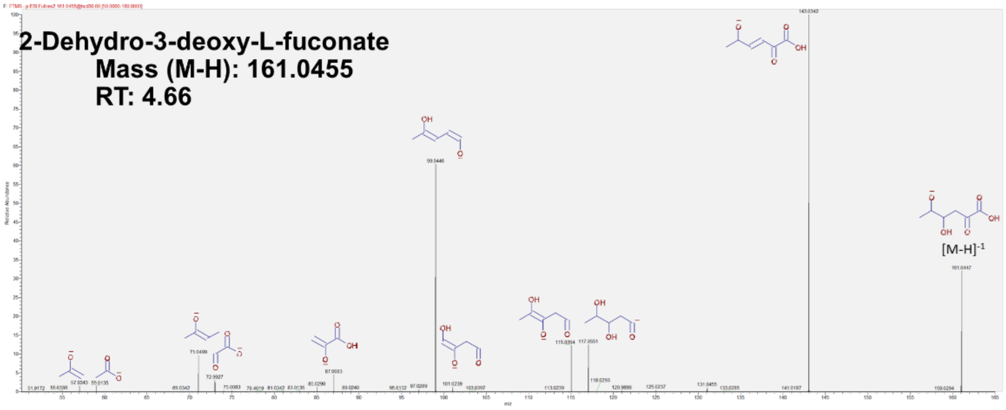

iii) LESA-MS/MS – Identified with confidence **level 3** by accurate mass and MS/MS that makes sense for the ID.

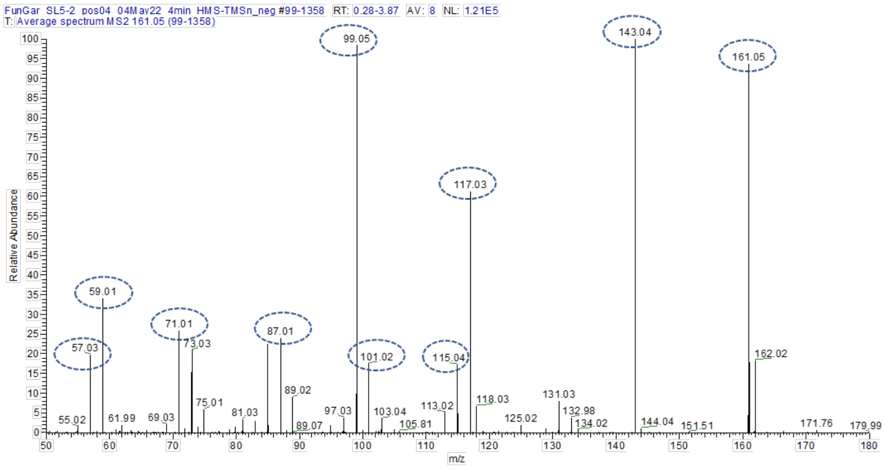

k) Glucose

i) **LC-IMS-MS/MS** – Identified with confidence **level 5** by accurate mass.
Caveat: Due to the low intensity of this metabolite, we used GC-MS (see below) to confidently identify glucose in the garden sample.

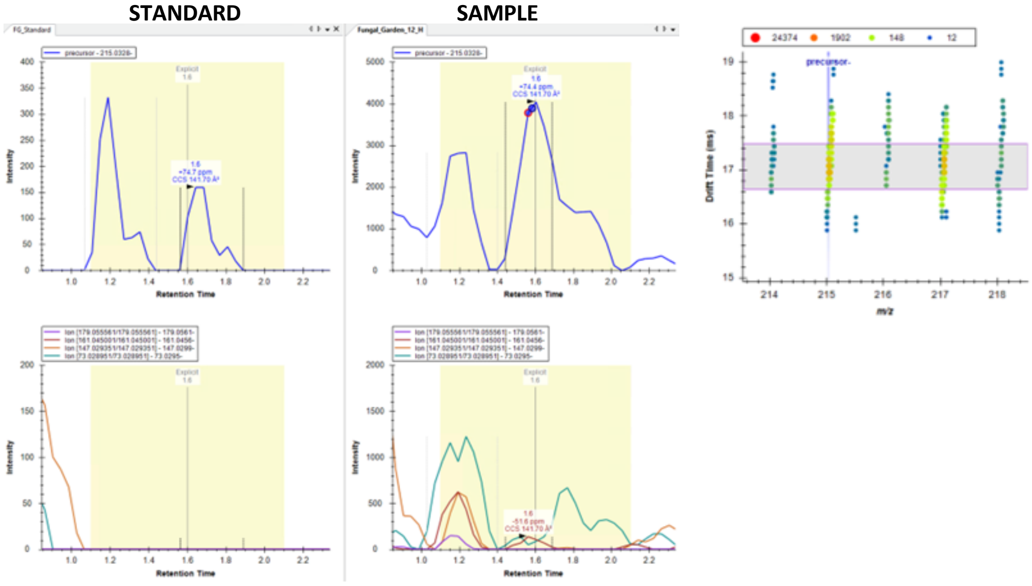

ii) GC-MS - Identified with confidence **level 1**. MS from in-house library. Retention index from sample 1607.4, retention index in standard: 1608.3. Fragments used to validate identification: 160, 205 and 319.

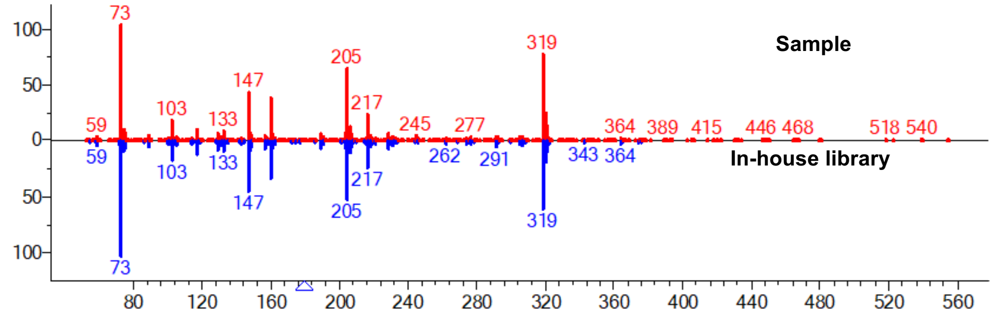

l) Glucose 6-phosphate

i) GC-MS – Identified with confidence **level 3**. MS from in-house library. Retention index from sample 2077.5 retention index in standard: 2049.0. Fragments used to validate identification: 299, 357 and 387. *Caveat: Due to the low intensity of this metabolite, we used LC-MS/MS (see below) to confidently identify glucose-6-phosphate in the garden sample.*

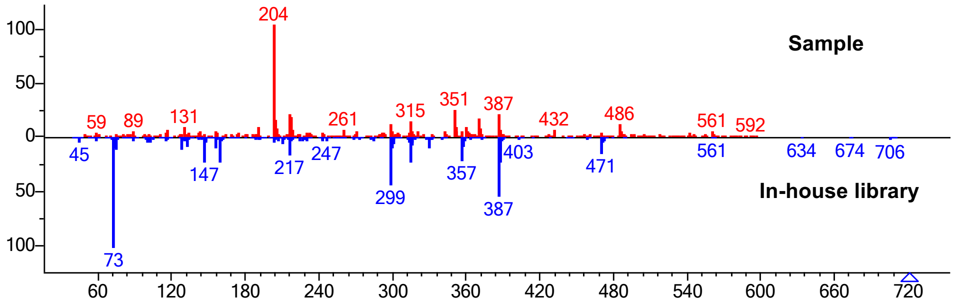

ii) LC-IMS-MS/MS – Identified with confidence **level 1** by accurate mass, RT, CCS, and MS/MS match with the standard.

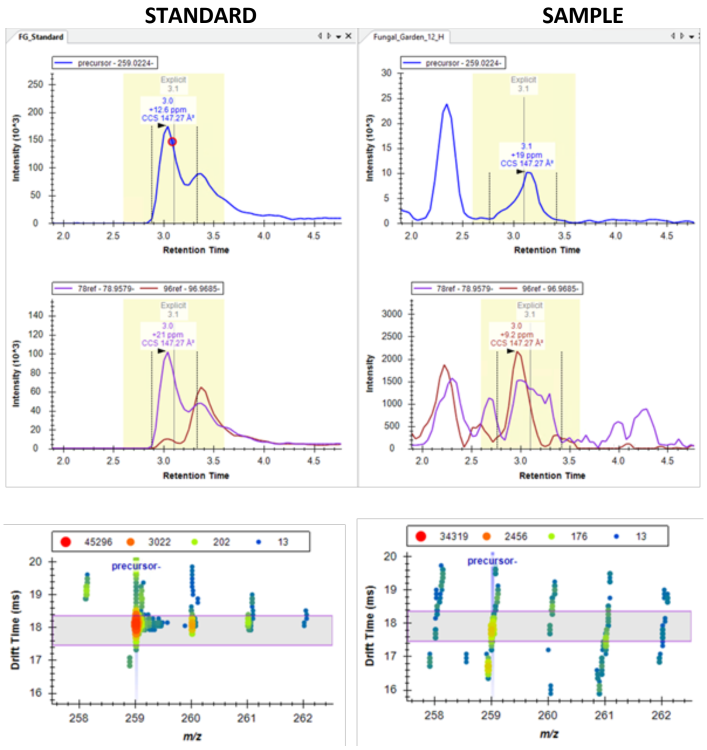

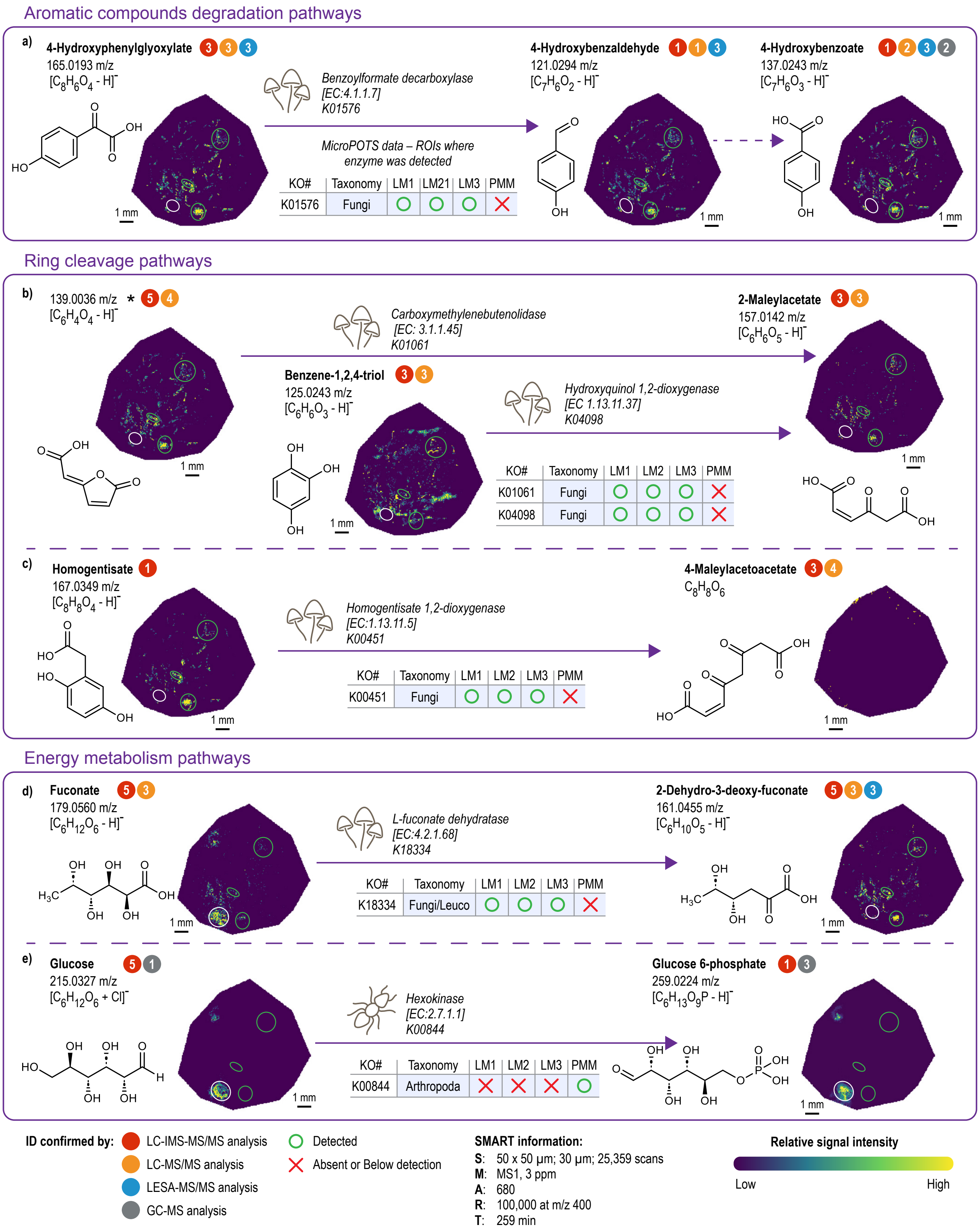

Supplementary Figure 3. Spatial multi-omics integration unravels complex catabolic pathways in the leaf-cutter ant fungal garden ecosystem. Metabolite MALDI-FTICR-MSI images visualize the relative abundance of each metabolite across Section 9 (yellow, highest intensity). These pathways include a) Conversion of 4-

hydroxyphenylglyoxylate by fungal benzoylformate decarboxylase. b) Two pathways of aromatic ring cleavage by fungus to 2-maleylacetate. c) Homogentisic pathway of homogentisate ring cleavage by fungus. d) Catabolic pathway of fuconate. e) Phosphorylation reaction of fuconate's mass isomer, glucose. MALDI-MSI information reported by SMART: S=step size; spot size; total scans; M=molecular confidence; A=annotations (METASPACE, KEGG, ≤ 20 FDR, $[M-H]^-[M+Cl]^-$); R=resolving power; T=time of acquisition. The three green circles and one white circle depict the LM (LM1, n=3; LM2, n=3; LM3, n=6) and PMM (PPM, n=3) ROIs, respectively. Complementary MicroPOTS enzyme identifications are specified above each reaction arrow with the table denoting if the enzyme was detected (O) or absent (X) in LM1, LM2, LM3 or PPM. A detailed list of enzymes with their corresponding peptides, taxonomic assignments, and ROI-specific localization, has been included in Supplementary Table 10. Colored circles next to the metabolite names indicate that metabolite identities were confirmed by different orthogonal techniques via tandem MS, included in **Supplementary Figure 2**. Numbers within each circle indicate metabolite identification metrics (i.e., 1 (high) → 5 (low)) as detailed in Supplementary Figure 5 and[1]. *$[C_6H_4O_4-H]^-$, this molecular formula matches to candidate molecules 4-Carboxymethylenebut-2-en-4-olide (the substrate of Carboxymethylenebutenolidase → 2-maleylacetate) and 5-Formyl-2-furoate in the KEGG database.

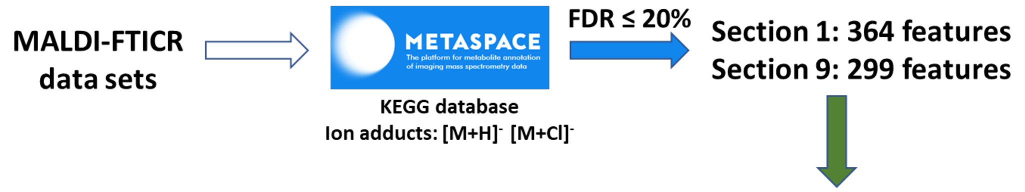

Metabolites validated by detection of paired enzymes:

Pathway	Metabolite	Localization	m/z	Section #1 - used in Fig 4 FDR (%)	Section #9 - used in Sup. Fig. 2 FDR (%)
a)	4-hydroxyphenylglyoxylate	LM1-3	165.0193	20	20
	4-hydroxybenzaldehyde	LM1-3	121.0294	20	20
	4-hydroxybenzoate	LM1-3	137.0243	10	10
b)	$[C_6H_4O_4 - H]^-$ *	LM1-3	139.0036	20	20
	Benzene-1,2,4-triol	LM2	125.0243	20	20
	2-maleylacetate	LM1-3	157.0142	20	20
c)	Homogentisate	LM1-3	167.0349	20	20
	4-maleylacetoacetate	/	not detected	/	/
d)	Fuconate	LM1-3, PMM	179.0560	50	50
	2-dehydro-3-deoxy-fuconate	LM1-3	161.0455	20	50
e)	Glucose	PMM	215.0327	20	20
	Glucose-6-phosphate	PMM	259.0224	10	10

$[C_6H_4O_4 -H]^-$, this molecular formula matches to candidate molecules, 4-Carboxymethylenebut-2-en-4-olide (the substrate of Carboxymethylenebutenolidase → 2-maleylacetate) and 5-Formyl-2-furoate, in the KEGG database.

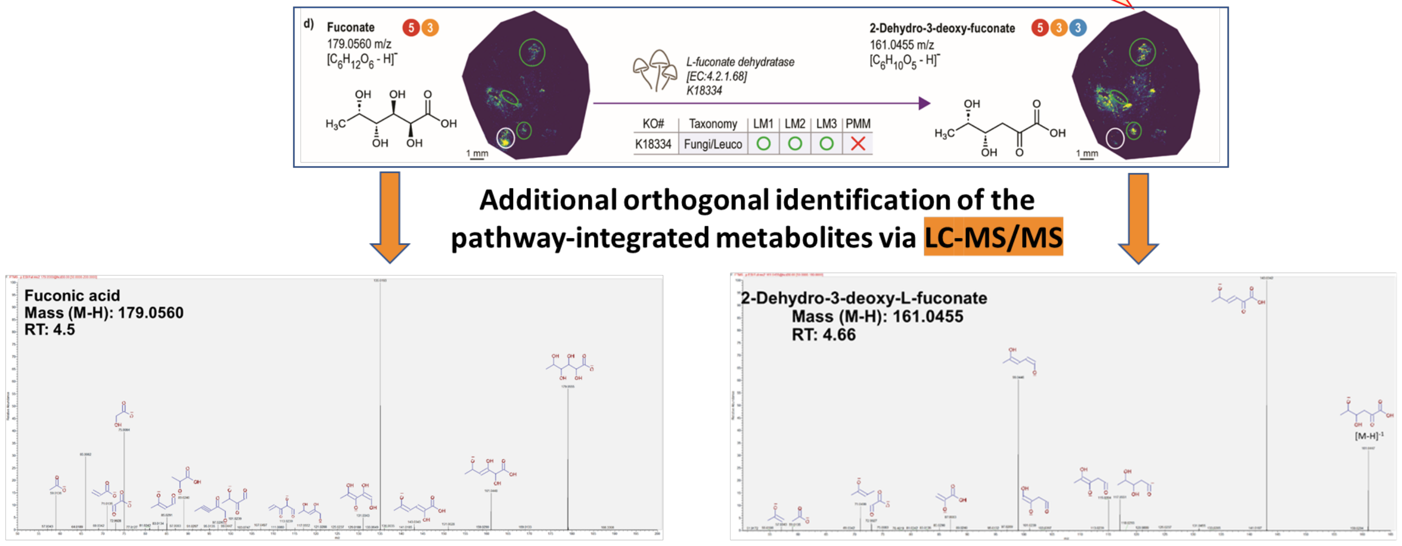

Supplementary Figure 4. A workflow with all the steps of the annotation and identification of fuconate in the MALDI-FTICR data.

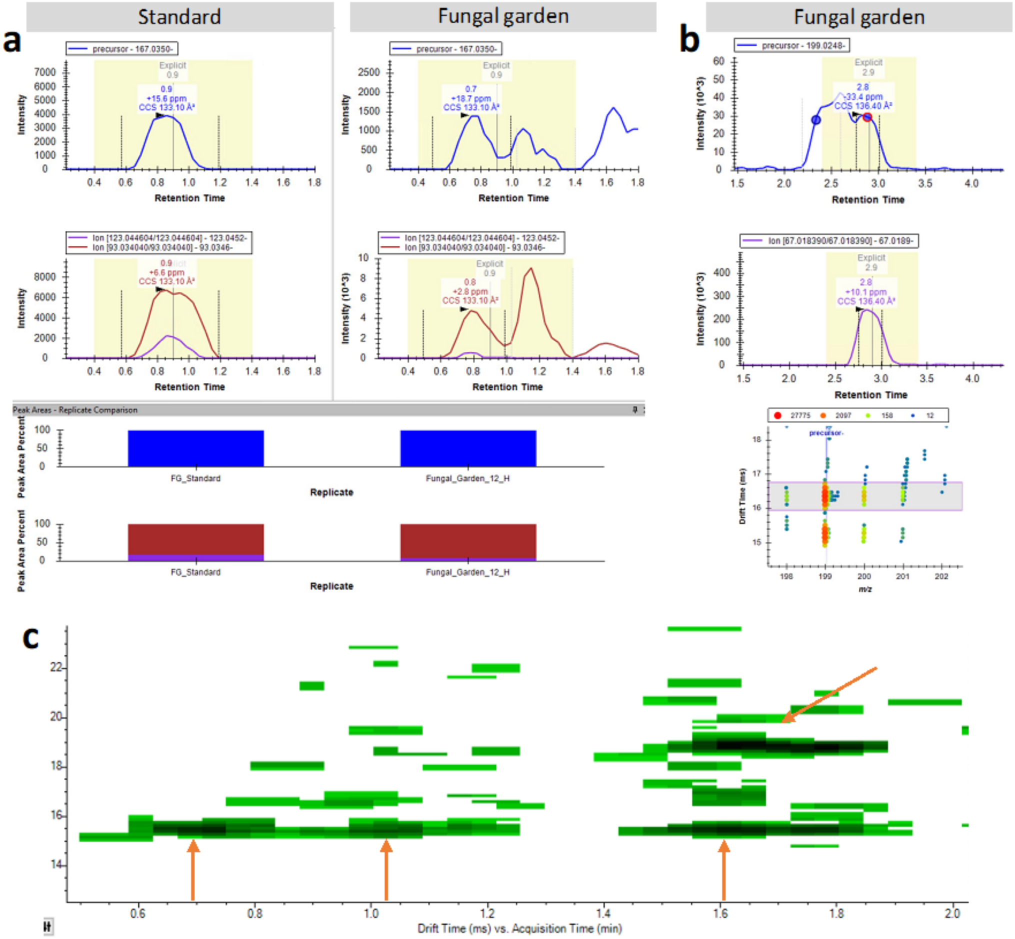

Supplementary Figure 5. Metabolites and isomers of the homogentisic pathway of homogentisate ring cleavage by fungus detected by LC-IMS-MS/MS. a) Extracted chromatograms of homogentisate detected from the standard and fungal garden sample. b) 4-Maleylacetoacetate detected signals of two isomers at 16.35 and 15.21 ms (136.4 and 127.1 CCS) in the fungal garden sample. c) 2D extracted ion chromatogram showing detected isomers for m/z 167.0350: three isomers at 15.77 ms (133.1 CCS) with RT 0.75 (homogentisate), 1.1 (putatively identified as vanillate) and 1.6 min (putatively identified as 4-hydroxymandelate), and one isomer at 19.39 ms (152 CCS) with RT 1.65 min.

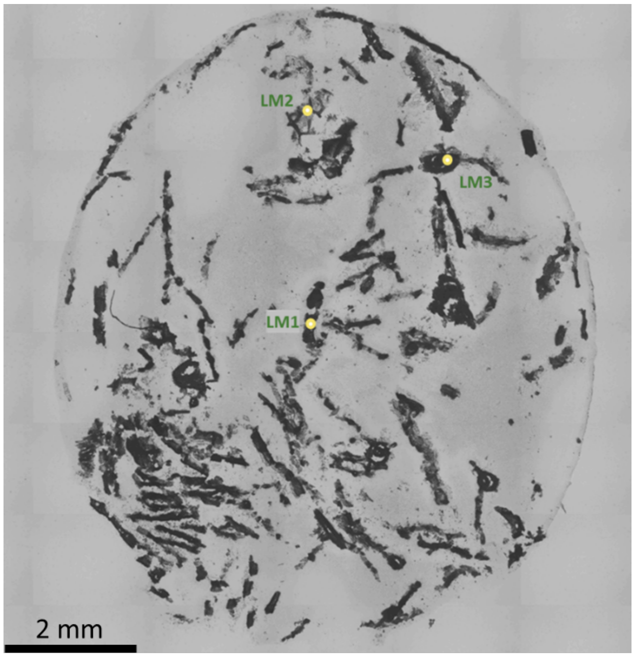

Supplementary Figure 6. Optical image of 20µm-thick fungal garden cryosection with mapped region of interests (ROIs) that were morphologically similar to the ones imaged using the Metabolome informed protein imaging (MIPI) approach. Three distinct ROIs similar to previously mapped lignin microhabitats LM1, LM2 and LM3 were sampled and directly infused into the mass analyzer.

Supplementary Table 6. **Metabolites confirmed by annotated LC-IMS-MS features from the fungal garden sample.** A list of targets with accurate masses for precursor and fragment ions was generated using predicted spectra by CFM-ID. Signals were extracted using Skyline from the LC-IMS-MS/MS data acquired in DIA mode from the fungal garden sample.

The column # of detected fragments indicates the count of fragments that had good peak shape similarity compared to the precursor.

Results are from the data acquired using the HILIC method from Bilbao et al.[2]

Putative/potential annotations of LC-IMS resolved isomers are denoted by "(*)".

Adduct	RT	CCS	Confirmed by standard	# detected fragments (MS/MS)	Metabolite	PubChem CID	Exact mass
M-H	4.8	150.2		2	4-hydroxyphenylglyoxylate	355	166.0266
M-H	0.8	119.4	Yes	2	4-hydroxybenzaldehyde	126	122.0368
M-H	1.0	122.1	Yes	4	4-hydroxybenzoate	54675830	138.0317
M-H	2.0	114.5		0	4-carboxymethylenebut-2-en-4-olide	120	140.0109
M-H	3.2	142.5		3	benzene-1,2,4-triol	10787	126.0317
M-H	4.0	140.5		5	2-maleylacetate	5280500	158.0215
M-H	0.8	133.1	Yes	2	homogentisate	780	168.0422
M-H	1.1	133.1		3	vanillate (*)	54675858	168.0422
M-H	1.6	133.1		0	4-hydroxymandelate (*)	5460261	168.0422
M-H	2.9	136.4		1	4-maleylacetoacetate	5280393	200.0321
M-H	0.9	129.3		0	fuconate	6857452	179.0556
M-H	0.7	138.6		0	2-dehydro-3-deoxy-fuconate	9548669	161.0449
[M+Cl]-	1.5	141.7	Yes	4	glucose	5793	180.0633
M-H	3.1	147.27	Yes	2	glucose 6-phosphate	5958	260.0297

Supplementary Table 7. Metabolites confirmed by annotated LC-MS/MS features from the fungal garden sample.

Adduct	Exact mass	Metabolite	PubChem CID	RT	MS/MS (Yes/No)
M-H	166.0266	4-hydroxyphenylglyoxylate	355	2.9	Yes
M-H	122.0368	4-hydroxybenzaldehyde	126	1.0	Yes
M-H	138.0317	4-hydroxybenzoate	54675830	2.7	Yes
M-H	140.0109	4-carboxymethylenebut-2-en-4-olide or 5-Formyl-2-furoate	120	multiple possibilities	No
M-H	126.0317	benzene-1,2,4-triol	10787	3.0	Yes
M-H	158.0215	2-maleylacetate	5280500	5.1	Yes
M-H	200.0321	4-maleylacetoacetate	5280393	multiple possibilities	No
M-H	179.0556	fuconate	6857452	4.6	Yes
M-H	161.0449	2-dehydro-3-deoxy-fuconate	9548669	4.7	Yes

Supplementary Table 8. Metabolites confirmed by annotated LESA-MS/MS features from the fungal garden sample.

Monoisotopic Mass	Metabolite	ROI of Detection	MetFrag Score
166.027	4-hydroxyphenylglyoxylate	LM1	0.9599
		LM2	0.9554
		LM3	0.9545
122.037	4-hydroxybenzaldehyde	LM1	0.9889
		LM2	0.9873
		LM3	1.0000
138.032	4-hydroxybenzoate	LM1	1.0000
		LM2	1.0000
		LM3	1.0000
162.053	2-dehydro-3-deoxy-fuconate	LM1	0.9713
		LM2	0.9818
		LM3	0.9969

Supplementary Table 9. Metabolites confirmed by annotated GC-MS features from the fungal garden sample.

Metabolite	PubChem CID	Retention Index (RI)	Fragments used for identification	Confirmed by standard
4-hydroxybenzoate	54675830	1315.4	193, 223, 267	Yes
glucose	5958	1608.3	160, 205, 319	Yes
glucose 6-phosphate	242	2049.0	299, 357, 387	Yes

1. Schymanski, E.L., *et al.* Identifying Small Molecules via High Resolution Mass Spectrometry: Communicating Confidence. *Environmental Science & Technology* **48**, 2097-2098 (2014).
2. Bilbao, A., *et al.* PeakDecoder enables machine learning-based metabolite annotation and accurate profiling in multidimensional mass spectrometry measurements. *Nature Communications* **14**, 2461 (2023).
