# Supplementary material for: Mapping microhabitats of lignocellulose decomposition by a microbial consortium
Source: Nat Chem Biol. 2024 Feb 1;20(8):1033–43. doi: 10.1038/s41589-023-01536-7 (PMC11288888; doi:10.1038/s41589-023-01536-7)
Supplement: Supplementary file 2 — Reporting Summary [file 41589_2023_1536_MOESM2_ESM.pdf]

nature portfolio

Corresponding author(s): Kristin Burnum-Johnson

Last updated by author(s): Dec 4, 2023

Reporting Summary

Nature Portfolio wishes to improve the reproducibility of the work that we publish. This form provides structure for consistency and transparency in reporting. For further information on Nature Portfolio policies, see our Editorial Policies and the Editorial Policy Checklist.

Statistics

For all statistical analyses, confirm that the following items are present in the figure legend, table legend, main text, or Methods section.

n/a	Confirmed	
☐	☒	The exact sample size (*n*) for each experimental group/condition, given as a discrete number and unit of measurement
☐	☒	A statement on whether measurements were taken from distinct samples or whether the same sample was measured repeatedly
☐	☒	The statistical test(s) used AND whether they are one- or two-sided<br>*Only common tests should be described solely by name; describe more complex techniques in the Methods section.*
☒	☐	A description of all covariates tested
☐	☒	A description of any assumptions or corrections, such as tests of normality and adjustment for multiple comparisons
☒	☐	A full description of the statistical parameters including central tendency (e.g. means) or other basic estimates (e.g. regression coefficient) AND variation (e.g. standard deviation) or associated estimates of uncertainty (e.g. confidence intervals)
☐	☒	For null hypothesis testing, the test statistic (e.g. *F*, *t*, *r*) with confidence intervals, effect sizes, degrees of freedom and *P* value noted<br>*Give P values as exact values whenever suitable.*
☒	☐	For Bayesian analysis, information on the choice of priors and Markov chain Monte Carlo settings
☒	☐	For hierarchical and complex designs, identification of the appropriate level for tests and full reporting of outcomes
☐	☒	Estimates of effect sizes (e.g. Cohen's *d*, Pearson's *r*), indicating how they were calculated

Our web collection on statistics for biologists contains articles on many of the points above.

Software and code

Policy information about availability of computer code

Data collection	MALDI MSI datasets were collected using FlexImaging (v 4.1).<br>Proteomics datasets were collected using Thermo Xcalibur software (v 4.0), Exactive software (v 2.8 SP1).
Data analysis	METASPACE (https://metaspace2020.eu) and SCiLS Lab (Version 2024a Core) were used for metabolite annotation and data visualization.<br>MSGFPlus (v2021.09.06), MASIC (Release 3.2.7901), CD-HIT (v4.8.1), Kofamscan (v1.2.0), JGI metagenome workflow (released on 18th May 2021), BLAST (v2.13.0+), Contig Annotation Tool (CAT, v5.2.3), and KEGGREST (v1.38.0) were used for metaproteomic data analyses.<br>R (version 3.2.1.) was used for statistical analyses. Metabolite Detector software (V2.520130704testing), Compound Discoverer (v3.3), PNNL-Preprocessor (v2020.07.24), IM-MS Browser (v.10.0, Agilent Technologies), CFM-ID (v4.0), Skyline (v.64.21.1.0.146), MetFrag (https://ipb-halle.github.io/MetFrag/).

For manuscripts utilizing custom algorithms or software that are central to the research but not yet described in published literature, software must be made available to editors and reviewers. We strongly encourage code deposition in a community repository (e.g. GitHub). See the Nature Portfolio guidelines for submitting code & software for further information.

Data

Policy information about availability of data

All manuscripts must include a data availability statement. This statement should provide the following information, where applicable:

- Accession codes, unique identifiers, or web links for publicly available datasets
- A description of any restrictions on data availability
- For clinical datasets or third party data, please ensure that the statement adheres to our policy

Publicly available MALDI MSI data:
Section 1:
https://metaspace2020.eu/annotations?db_id=374&prj=e6a0f848-d586-11ed-afbb-1b53f1cbcdef&ds=2021-09-20_21h49m04s&fdr=0.2&sort=fdr_msm&sections=5&row=2

Section 9:
https://metaspace2020.eu/annotations?db_id=374&prj=e6a0f848-d586-11ed-afbb-1b53f1cbcdef&ds=2021-09-21_23h56m53s&fdr=0.2&row=2

Raw proteomics data:
Mass Spectrometry Interactive Virtual Environment (MassIVE) (https://massive.ucsd.edu) with accession number MSV000091701. The protein database was deposited at Zenodo (https://zenodo.org/record/7826514#.ZEw5V-zMLtV) with DOI: 10.5281/zenodo.7826514.

Human research participants

Policy information about studies involving human research participants and Sex and Gender in Research.

Reporting on sex and gender	N/A
Population characteristics	N/A
Recruitment	N/A
Ethics oversight	N/A

Note that full information on the approval of the study protocol must also be provided in the manuscript.

Field-specific reporting

Please select the one below that is the best fit for your research. If you are not sure, read the appropriate sections before making your selection.

☒ Life sciences ☐ Behavioural & social sciences ☐ Ecological, evolutionary & environmental sciences

For a reference copy of the document with all sections, see nature.com/documents/nr-reporting-summary-flat.pdf

Life sciences study design

All studies must disclose on these points even when the disclosure is negative.

Sample size	No sample-size calculation was performed. Biological replicates in this study were independent microhabitats where depth profiling was performed. All metaproteomic experiments to characterize lignin pathways were performed in at least three independent section replicates. We chose the number of replicates to be 3 in order to ensure variances could be estimated and independence of missing data statistics could be calculated.
Data exclusions	No data were excluded from the analyses.
Replication	The reproducibility of the metabolomics data was tested by the MALDI MSI analysis using two (108 µm apart) cryosections from the embedded fungal garden sample. For proteomic depth profiling, sections in close proximity to each of the two MALDI-imaged fungal garden sections were obtained. In the adjacent fungal garden sections, we were able to visually distinguish and analyze, Lignin Microhabitat 1, 2, and 3 (LM1, LM2, LM3) and Primary Metabolites Microhabitat (PPM). As LM3 was a larger microhabitat along the depth gradient, six sections corresponding to LM3 were collected and independently processed in contrast to three sections for the other three microhabitats (i.e., LM1, LM2, and PMM) to demonstrate the reproducibility in metaproteomic data. All replication attempts were successful.
Randomization	Each microhabitat was treated as an experimental group where samples were processed and analyzed using randomized orders.
Blinding	Investigators were not blinded to group allocation during data collection or analysis. Blinding is not common for the experiments performed in similar exploratory studies.

Reporting for specific materials, systems and methods

We require information from authors about some types of materials, experimental systems and methods used in many studies. Here, indicate whether each material, system or method listed is relevant to your study. If you are not sure if a list item applies to your research, read the appropriate section before selecting a response.

Materials & experimental systems

n/a	Involved in the study
☒	☐ Antibodies
☒	☐ Eukaryotic cell lines
☒	☐ Palaeontology and archaeology
☐	☒ Animals and other organisms
☒	☐ Clinical data
☒	☐ Dual use research of concern

Methods

n/a	Involved in the study
☒	☐ ChIP-seq
☒	☐ Flow cytometry
☒	☐ MRI-based neuroimaging

Animals and other research organisms

Policy information about studies involving animals; ARRIVE guidelines recommended for reporting animal research, and Sex and Gender in Research

Laboratory animals	Fungal garden samples were collected from Atta cephalotes colonies maintained in the laboratory. These colonies were reared on a mix of red maple (Quercus rubra) and red oak (Acer rubrum) leaves. Atta colonies were collected in 2018.
Wild animals	The study didn't involve wild animals.
Reporting on sex	This was not relevant for our study hence it was not collected.
Field-collected samples	The study did not involve samples collected from the field.
Ethics oversight	No ethical approval or guidance was required.

Note that full information on the approval of the study protocol must also be provided in the manuscript.
